# A Facile Eutectic Strategy for Scalable, Leakage‐Free Thermochromic Phase‐Change Composites Enabling Smart Temperature Labels and Secure Data Encryption

**DOI:** 10.1002/advs.202519934

**Published:** 2025-11-12

**Authors:** Shiliang Zhou, Qianyi Zhang, Huan Liu, Zhiqiang Qian, Xiaodong Wang

**Affiliations:** ^1^ State Key Laboratory of Organic–Inorganic Composites Beijing University of Chemical Technology Beijing 100029 China; ^2^ Key Laboratory of Green and High‐End Utilization of Salt Lake Resources Qinghai Institute of Salt Lakes Chinese Academy of Sciences Qinghai Provincial Key Laboratory of Resources and Chemistry of Salt Lakes Xining Qinghai 810008 China

**Keywords:** eutectic strategy, phase change materials, shape stability, temperature indication, thermochromic performance

## Abstract

Solid–liquid phase change materials (PCMs), when used as solvents in organic thermochromic composite systems, often suffer from leakage issues, which degrade their reversible color‐changing capability. Although encapsulation and porous composite shape‐stabilization techniques can enhance the structural integrity of PCMs, these methods are often complex, limited to lab‐scale production, and costly. Hereby, a facile eutectic strategy is presented to develop leakage‐free PCMs for stabilizing organic thermochromic composites by integrating organic lauric acid (LA) and inorganic sodium acetate trihydrate (SAT). Molecular dynamics simulations reveal significantly enhanced intermolecular interactions between SAT and LA compared to those between LA molecules. These strengthened intermolecular interactions endow the SAT/LA eutectic with excellent shape stability, preventing leakage and phase separation. Benefiting from the superior properties of the SAT/LA eutectic, the resulting thermochromic composites exhibit outstanding shape stability, cyclically reversible thermochromic performance, and considerable latent heat capacity. Leveraging these advantages, applications in temperature indicators, thermally regulated quick response codes, information encryption, and data storage/disguise are successfully demonstrated. This work, guided by molecular dynamics simulations, offers a promising approach to designing industrial‐scale, cost‐effective, shape‐stabilized thermochromic materials for temperature indication and information encryption.

## Graphical abstract

A facile eutectic strategy is developed, guided by molecular dynamics simulations, to create leakage‐free phase change materials from lauric acid and sodium acetate trihydrate. These organic–inorganic eutectic composites effectively stabilize thermochromic dyes, yielding materials with exceptional shape stability and cyclically reversible color change. This enables advanced applications in smart labels, information encryption, and thermal data storage.

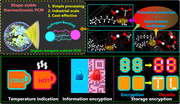

## 1 Introduction

Thermochromic materials are stimuli‐responsive substances that exhibit reversible or irreversible color changes in response to temperature variations within specific ranges. Since their first reported discovery in 1938, various thermochromic materials have been developed for diverse applications, including thermal‐regulating fabrics,^[^ ^1^, ^2^ ^]^ wearable temperature sensors,^[^ ^3^, ^4^, ^5^ ^]^ smart building materials,^[^ ^6^, ^7^ ^]^ adaptive windows,^[^ ^8^, ^9^, ^10^, ^11^ ^]^ cold chain transports,^[^ ^12^ ^]^ and information security systems.^[^ ^13^, ^14^, ^15^, ^16^ ^]^ These materials can be categorized by their operational temperature ranges: low‐temperature (< 100 °C), medium‐temperature (100−600 °C), and high‐temperature (> 600 °C) types.^[^ ^17^ ^]^ Inorganic thermochromic materials, typically medium‐ or high‐temperature variants, operate through mechanisms such as crystal phase transitions, electron transfer, or ligand geometry transformations, making them suitable for harsh environments. In contrast, organic thermochromic materials function at lower temperatures and find widespread use in healthcare, consumer products, and wearable technologies.^[^ ^18^, ^19^, ^20^ ^]^ Organic thermochromic systems typically consist of three key components: an electron donor (determining color), an electron acceptor (controlling color depth), and a solvent (governing transition temperature). The system's properties can be precisely tuned by selecting appropriate electron donors, adjusting acceptor concentrations, and modifying solvent composition with different melting points (*T*_m_).^[^ ^21^, ^22^ ^]^ For example, crystal violent lactone (CVL) and *6′*‐(diethylamino)‐*1′,2′*‐benzofluorane (DEABPF) demonstrate characteristic blue‐to‐white and pink‐to‐white transitions, respectively, when paired with weak acid acceptors like bisphenol A (BPA). Solid‐liquid phase change materials (PCMs) such as *1*‐dodecanol (*T*_m_ = 24 °C) and cetyl alcohol (*T*_m_ = 46 °C) are commonly employed as solvents. Despite their promising reversible thermochromic behavior ^[^ ^23^, ^24^, ^25^ ^],^ organic PCM‐based systems face a critical technical challenge that must be resolved before practical implementation: leakage and shape instability.

**Figure 1:**
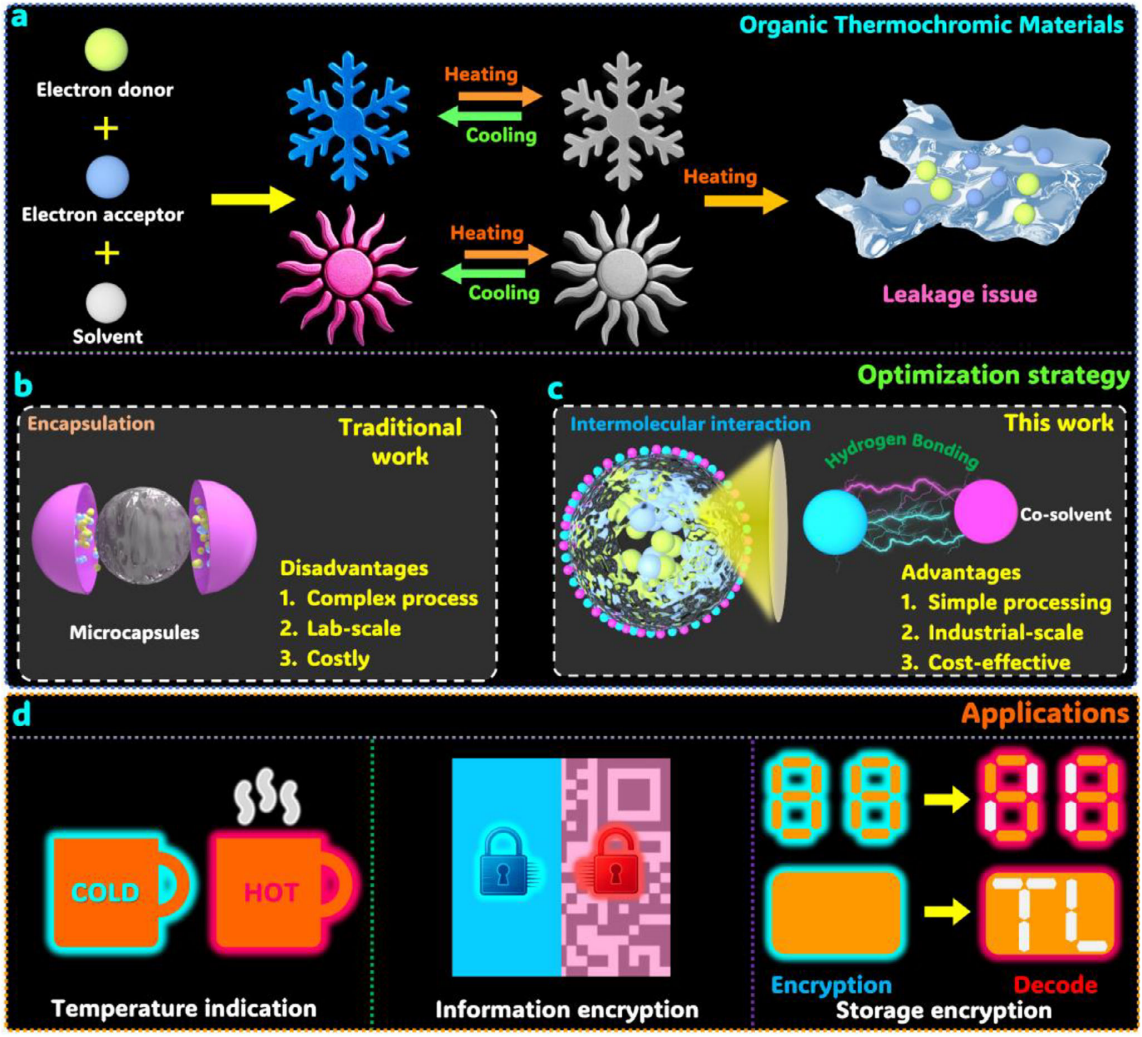
a) Thermochromic behavior and inherent leakage issue of organic thermochromic materials. b) Limitations of conventional thermochromic microcapsules. c) Key advantages of the developed shape‐stabilized thermochromic composite. d) Potential application domains for the shape‐stabilized thermochromic composite.

Solid–liquid PCMs used as solvents in organic thermochromic systems face significant leakage issues during solid–liquid phase transitions, which compromise their reversible color‐changing capability (**Figure** **1**a). To address this challenge, shape‐stabilization techniques involving encapsulation within protective shell materials have been widely adopted. In typical encapsulation approaches, solid–liquid PCMs are confined within organic or inorganic shells using various polymerization methods, including miniemulsion polymerization, in situ polymerization, interfacial polymerization, solvent evaporation, and suspension polymerization.^[^ ^26^, ^27^, ^28^, ^29^ ^]^ These shell materials serve as robust physical barriers, effectively containing the PCMs within defined spaces and preventing leakage during phase transitions. Due to their demonstrated effectiveness, thermochromic micro/nano‐encapsulated PCMs have garnered significant research attention and commercial interest.^[^ ^30^, ^31^ ^]^ Researchers have developed diverse thermochromic microcapsule systems featuring reversible color changes by encapsulating organic thermochromic components within organic shells [e.g., poly(methyl methacrylate) and poly(methyl methacrylate‐co‐methacrylic acid)],^[^ ^32^, ^33^ ^]^ inorganic shells (e.g., SiO_2_ and CaCO_3_),^[^ ^34^, ^35^ ^]^ and organic/inorganic hybrid shells.^[^ ^36^ ^]^ These encapsulation systems consistently demonstrate excellent shape stability, durability, and reversible thermochromic performance, confirming encapsulation as a highly effective strategy for developing shape‐stabilized organic thermochromic materials.

Despite enhancing PCM structural integrity and achieving some commercial success, encapsulation techniques often involve complex, costly processing that is difficult to scale beyond the lab. These factors hinder their adoption in large‐scale, cost‐sensitive applications for thermochromic microcapsules (Figure 1b). Developing cost‐effective shape‐stabilization techniques compatible with scalable manufacturing remains a significant challenge for practical applications. As an alternative approach, porous material‐based stabilization through impregnation offers a relatively simpler process compared to encapsulation techniques.^[^ ^37^ ^]^ The high specific surface area, abundant pore structures, and low density of porous materials enable effective PCM stabilization through surface tension effects, capillary forces, and hydrogen bonding interactions.^[^ ^38^, ^39^ ^]^ Nevertheless, this method requires pre‐processing of porous supports – for instance, freeze‐drying typically demands several days to prepare suitable porous matrices. Furthermore, commonly used PCM solvents for organic thermochromic materials (e.g., *1*‐tetradecanol, ethyl myristate, and *n*‐butyl stearate) remain prohibitively expensive for large‐scale adoption. Another promising strategy involves chemically incorporating PCM molecules into polymer chains to create solid–solid PCMs.^[^ ^40^, ^41^ ^]^ While the strong covalent bonds in these systems effectively restrict molecular chain mobility and prevent leakage during phase transitions, the resulting materials cannot function as liquid solvents for thermochromic components, limiting their applicability in organic thermochromic systems.

While chemical bonding interactions have not fully resolved the challenges facing organic thermochromic materials for large‐scale applications, they have demonstrated the potential of molecular interactions for achieving shape‐stabilized PCMs. Building on this concept, we propose a general eutectic strategy to develop shape‐stabilized solid–liquid PCMs through intermolecular interactions, enabling the construction of leakage‐free thermochromic phase‐change composites for reversible temperature indication and information encryption. Eutectic PCMs, formed by combining two or more PCM components, offer tunable phase‐change enthalpies and transition temperatures, making them adaptable for diverse applications, including building thermal regulation, biomedical technologies, battery thermal management, and solar‐thermal energy systems.^[^ ^42^, ^43^, ^44^, ^45^ ^]^ Although these multicomponent systems still face leakage challenges similar to single‐component PCMs, eutectic mixtures demonstrate enhanced thermal and structural stability through strengthened intermolecular interactions.^[^ ^46^, ^47^ ^]^ This suggests that proper component selection can yield shape‐stabilized eutectic PCMs without requiring additional stabilization methods.

In this work, we selected low‐cost organic lauric acid (LA) and inorganic sodium acetate trihydrate (SAT) as our eutectic system components. While both materials exhibit leakage in their pure states, molecular dynamics simulations reveal that a 4:6 mass ratio SAT/LA eutectic forms sufficiently strong intermolecular interactions to prevent leakage. Experimental results confirm this leakage‐free behavior, enabling direct application in thermochromic systems. Compared to conventional approaches, our developed thermochromic system offers three key advantages: 1) simplified processing, 2) industrial‐scale feasibility, and 3) cost‐effectiveness (Figure 1c). These benefits translate to superior reversible thermochromic performance suitable for temperature indication, information encryption, and secure data storage applications (Figure 1d). By innovatively combining theoretical analysis with experimental validation to integrate self‐stabilized PCMs into thermochromic systems, this study establishes a new paradigm for developing advanced, economically viable thermochromic materials ready for large‐scale implementation.

## 2 Results and Discussion

### 2.1 Interaction Energy of SAT/LA Eutectics

**Figure 2:**
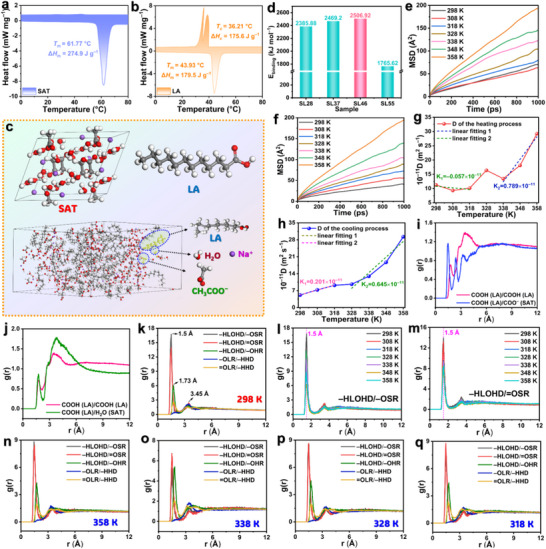
DSC thermograms of a) pure SAT and b) LA. c) Representative equilibrated configuration of the SL46 simulation model. d) Calculated binding energy (*E*_binding_) values for SAT/LA mixture systems. MSD curves for SL46 during e) heating and f) cooling cycles (298–358 K, 10 K intervals). Evolution of the self‐diffusion coefficient (D) for SL46 during g) heating and h) cooling. i) RDFs between acetate groups in LA‐LA and LA‐SAT pairs. j) RDFs between acetate groups in LA/LA and between acetate (LA) and H_2_O (SAT) pairs. k) RDFs for specific hydrogen bonding interactions at 298 K during heating. RDFs for l) –HLOHD/–OSR and m) –HLOHD/ = OSR interactions at selected heating temperatures. RDFs for different types of hydrogen bonding interactions at n) 358, o) 338, p) 328, and q) 318 K during cooling.

SAT and LA are typical solid–liquid PCMs offering high latent heat capacity, suitable phase‐change temperatures, and low cost for low‐to‐medium temperature applications. Differential scanning calorimetry (DSC) shows that SAT has a melting enthalpy (Δ*H*_m_) of 274.9 J g^−1^ and a melting temperature (*T*_m_) of 61.77 °C (**Figure** **2**a), while LA exhibits values of 179.5 J g^−1^ and 43.93 °C, respectively (Figure 2b). Intermolecular interactions effectively mitigate leakage in solid–liquid PCMs.^[^ ^48^ ^]^ Given the chemical characteristics of SAT and LA, hydrogen bonding can occur in their mixture. To identify an optimal SAT/LA mass ratio for molecular simulation, we predicted the theoretical eutectic composition using thermal properties from DSC (Table S2, Supporting Information). As illustrated in Figure S1 (Supporting Information), the Schröder‐Van Laar equation yielded a eutectic mole fraction of LA of 0.687 (corresponding SAT/LA mass ratio = 1:3.23).^[^ ^49^ ^]^ Based on this, SAT/LA mass ratios of 2:8, 3:7, 4:6, and 5:5 (denoted SL28, SL37, SL46, SL55) were selected for binding energy evaluation. Snapshots of the simulation systems are shown in Figure 2c and Figure S2 (Supporting Information) (simulation details in Section S1, Supporting Information). Prior to production simulations, we validated the stability and authenticity of our simulation systems. The SL46 eutectic system, used as a representative, was equilibrated under an NVT ensemble at 298 and 358 K. As shown in Figure S3a (Supporting Information), potential energy, kinetic energy, and non‐bonded energy exhibit stable fluctuations, resulting in a converged total energy. The real‐time temperature fluctuates within 5% of the target value during equilibration (Figure S3b, Supporting Information). At 298 K, the average temperature is 297.8 K, closely matching the target (298 K). Density was further used to validate correspondence with experimental conditions. The average density at 298 K is 1.027 g cm^−3^ (Figure S3c, Supporting Information), consistent with the theoretical value of 1.044 g cm^−3^. Similarly, at 358 K, the system achieved stable total energy, an average temperature of 358.3 K, and an average density of 0.986 g cm^−3^ (Figure S4, Supporting Information). These results confirm that both systems reached equilibrium and were properly configured for subsequent simulations. Figure 2d presents the binding energy values for the SAT/LA mixtures. Among these systems, SL46 exhibits the highest binding energy value. The calculated binding energy values are 2385.88 kJ mol^−1^ (SL28), 2469.20 kJ mol^−1^ (SL37), 2506.92 kJ mol^−1^ (SL46), and 1765.62 kJ mol^−1^ (SL55). The significantly higher binding energy for SL46 indicates stronger intermolecular interactions within this system. Consequently, SL46 was selected as the representative sample for further analysis of phase‐change behavior and intermolecular interactions.

Mean square displacement (MSD) quantifies changes in particle positions over time and serves as a probe for molecular diffusion, transport, and binding states. Figure 2e,f presents the MSD curves for heating and cooling cycles between 298 and 358 K in 10 K intervals. At lower temperatures, SAT and LA molecules reside primarily in a stable crystalline state, exhibiting constrained molecular mobility. This results in smaller MSD values and sluggish growth. As temperature increases, enhanced thermal energy disrupts intra‐system hydrogen bonds and van der Waals forces, promoting a gradual transition toward a liquid state and significantly increasing molecular migration. This behavior aligns with the Arrhenius equation, which predicts an exponential relationship between diffusion coefficient and temperature.^[^ ^50^ ^]^ Notably, the MSD curve shows particularly slow growth at 308 K during heating, indicating restricted molecular motion. The self‐diffusion coefficient, a key indicator of phase transitions,^[^ ^51^ ^]^ exhibits non‐monotonic variations during heating (Figure 2f). This irregularity likely arises from initial incomplete mixing of SAT and LA, leading to structural inhomogeneity. The slope of the self‐diffusion coefficient inflects at 318 and 338 K, corresponding to the melting points of LA and SAT, respectively. These simulated transition temperatures align well with the *T*_m_ values obtained from DSC thermograms (Figure 2a,b), demonstrating agreement between simulation and experiment. In contrast, the cooling process exhibits a smoother and more regular self‐diffusion coefficient curve (Figure 2g). This suggests that after sufficient thermal equilibration, SAT and LA form a stable eutectic system, responding uniformly to decreasing temperature. Significantly, only one inflection point appears near 318 K during cooling, consistent with the phase transition of the SL46 eutectic mixture and indicating its formation.

### 2.2 Evolution of Intermolecular Interactions

Given the molecular characteristics of SAT and LA, the primary intermolecular interactions arise from acetate/acetate and acetate/H_2_O interactions between SAT and LA. Figure 2i presents the radial distribution functions (RDFs) for acetates in SAT/LA and LA/LA pairs. An RDF describes the probability *g(r)* of finding a neighboring molecule at a radial distance (*r*), providing insights into the nature of physical or chemical interactions between molecules.^[^ ^51^ ^]^ In Figure 2i, the first RDF peaks for SAT/LA and LA/LA appear at radial distances of 1.45 and 1.79 Å, respectively. Both distances are below 3.5 Å, indicative of hydrogen bonding interactions.^[^ ^52^ ^]^ Notably, the first peak intensity is higher for SAT/LA than for LA/LA, suggesting stronger and more compact hydrogen bonding between acetates in SAT/LA. Additionally, Figure 2j reveals that the RDF peak intensity for acetate/H_2_O interactions in LA/SAT exceeds the acetate interactions in LA/LA, further confirming the enhanced heteromolecular interactions in the LA/SAT system contributed by H_2_O. This result indicates that H_2_O in SAT can act as a bridge to form hydrogen bonds with both the acetate groups of SAT and the hydroxyl group of LA, which is crucial for forming the continuous network for the eutectic system that prevents phase separation.

To further investigate hydrogen bonding in the SL46 eutectic system, we performed RDF analysis on molecular dynamics trajectories at various temperatures during heating and cooling. Each trajectory comprised 1001 frames, with the latter 501 frames (501–1001) selected for analysis. We examined five hydrogen bonding types, categorized by their acceptors and donors:

- **Hydrogen bond acceptors**: −O in SAT acetate (−OSR), = O in SAT acetate ( = OSR), −O in SAT H_2_O (−OHR), −O in LA (−OLR), and = O in LA ( = OLR).
- **Hydrogen bond donors**: −H in SAT H_2_O (−HHD) and −H in LA (−HLOHD).

The resulting hydrogen bond interactions include −HLOHD/–OSR, −HLOHD/ = OSR, −HLOHD/−OHR, −OLR/−HHD, and = OLR/−HHD. Figure 2k and Figure S5 (Supporting Information) display the RDFs for these hydrogen bonds at different heating temperatures. The primary RDF peaks exhibit three distinct regions, reflecting variations in interaction strength. The RDF peaks for −HLOHD/−OSR and −HLOHD/ = OSR appear near 1.5 Å, consistent with the typical bond length of strong carboxyl‐hydroxyl hydrogen bonds.^[^ ^53^ ^]^ In contrast, the peak for −HLOHD/−OHR shifts slightly to 1.73 Å, likely due to the dynamic behavior and steric hindrance of water molecules, which increase the average hydrogen bond distance.^[^ ^54^ ^]^ The peaks for −OLR/−HHD and = OLR/−HHD are notably broader and centered at 3.45 Å, suggesting that water molecules may act as bridges in the SAT/LA system, facilitating extended hydrogen‐bonded networks rather than forming direct, strong hydrogen bonds. In this case, the diversity of hydrogen bond types and local molecular environments collectively contributes to the observed variations in RDF peak positions.

During heating, the dominant intermolecular interaction in the system undergoes dynamic changes. While the −HLOHD/−OSR interaction remains predominant at most temperatures, −HLOHD/ = OSR becomes the strongest interaction at two specific temperatures: 318 and 348 K. This shift suggests that the SL46 system undergoes significant microstructural rearrangement or a phase transition as the temperature approaches or reaches the *T*_m_'s of LA and SAT. We hypothesize that at these critical temperatures, intensified thermal motion of molecules, increased flexibility of LA segments, and structural changes in SAT alter the local electron density of = OSR, enhancing its ability to compete for hydrogen bonding with −HLOHD. This temporary strengthening of −HLOHD/ = OSR may reflect thermodynamic rebalancing in the phase transition region, where intermolecular forces reconfigure to adapt to a new equilibrium state for the SL46 system. Figure 2l,m illustrates the temperature‐dependent evolution of the two key hydrogen bonds, −HLOHD/−OSR and −HLOHD/ = OSR. At 298 K, both interactions exhibit high RDF peak intensities, consistent with the tightly packed molecular arrangement and strong interactions in the solid state. As temperature increases, their intensities generally decline. Notably, the −HLOHD/−OSR interaction shows two distinct weakening‐to‐strengthening fluctuations, with minima near 318 and 348 K. Conversely, the −HLOHD/ = OSR interaction strengthens at these same temperatures. This alternating behavior indicates a competitive relationship between −OSR and = OSR as hydrogen bond acceptors. Near the phase transition points, = OSR temporarily dominates, while −OSR remains the primary contributor at other temperatures. These observations highlight the dynamic equilibrium of intermolecular forces in SL46 during heating, where structural transitions at critical temperatures drive shifts in hydrogen bonding preferences.

During cooling, the RDF peak positions remain consistent with those observed during heating (Figure 2n–q; Figure S6a–c, Supporting Information), confirming that the same types of hydrogen bonds persist. However, the evolution of dominant interactions is completely different. At the initial cooled state (358 K), intermolecular forces become more homogenized, with −HLOHD/−OSR showing relative dominance. Upon cooling to 338 K, the −HLOHD/ = OSR interaction strengthens, suggesting molecular rearrangement toward a more ordered structure in preparation for phase transition. At 328 K, the intensities of −HLOHD/−OSR and −HLOHD/ = OSR reach near equilibrium, reflecting a competitive balance during phase transition. Further cooling to 318 K significantly intensifies −HLOHD/ = OSR, marking the completion of a critical phase transition and the formation of a stable solid‐state arrangement. This behavior aligns with the predicted phase transition of the SL46 system, as identified through DSC analysis.

Figure S6d,e (Supporting Information) provides detailed insights into the temperature‐dependent evolution of the two hydrogen bonding interactions during cooling. The −HLOHD/–OSR interaction exhibits relatively high intensity at 358 K but weakens upon cooling to 338 K, reaching a plateau near 318 K. As the temperature further decreases to 298 K, the interaction strength gradually recovers. In contrast, the −HLOHD/ = OSR interaction shows relatively low intensity at 358–348 K. However, as the system approaches and enters the phase transition zone (338–318 K), its strength increases progressively, underscoring its critical role in driving the phase transition. Following the completion of the phase transition, the interaction intensity decreases slightly with further cooling. These results clearly demonstrate the dynamic evolution and competitive relationship between these intermolecular forces during the liquid–solid phase transition. The −HLOHD/ = OSR interaction plays a pivotal role in driving and completing crystallization within the phase transition temperature range, while the −HLOHD/−OSR interaction dominates in both the pre‐transition liquid and post‐transition solid states. The synergistic yet competitive interplay between these two interactions constitutes the fundamental driving force behind the phase transition in the SL46 system.

**Figure 3:**
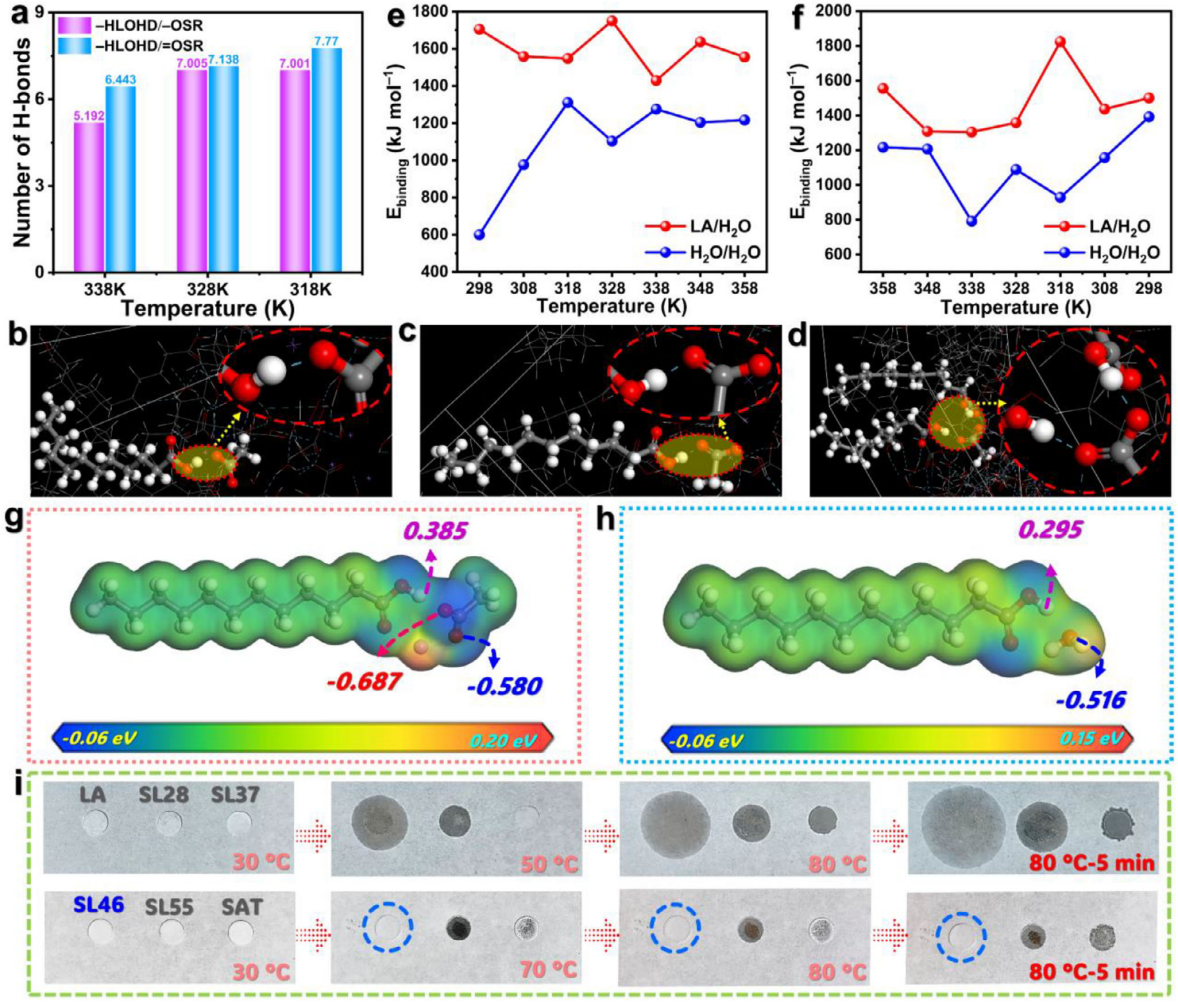
a) Average hydrogen bond numbers of the SAT/LA eutectic system at different cooling temperatures. Molecular schematic illustrations of hydrogen bonding configurations for b) –HLOHD/–OSR interaction, c) –HLOHD/ = OSR interaction, and d) cooperative interaction between −HLOHD/−OSR and –HLOHD/ = OSR. *E*_binding_ comparison between LA/H_2_O and H_2_O/H_2_O interactions during e) heating and f) cooling cycles. MEP and charge density analysis for g) LA/SAT and h) LA/H_2_O interfaces. i) Digital photographs of pure LA, SAT, and their eutectics during heating.

Given the crucial role of −HLOHD/−OSR and −HLOHD/ = OSR interactions in the 338–318 K temperature range during cooling, we conducted a statistical analysis of their average hydrogen bond formation numbers (**Figure** **3**a). Quantitative analysis reveals that at 338 K, the −HLOHD/ = OSR interaction forms significantly more hydrogen bonds (6.433) than −HLOHD/−OSR (5.129), indicating the dominant role of = OSR‐mediated interactions in initiating molecular rearrangement and subsequent phase transition. As the temperature decreases to 328 K, both interactions increase in frequency (7.318 for −HLOHD/ = OSR versus 7.005 for −HLOHD/−OSR), reaching near‐equilibrium values that reflect a dynamic balance between competing hydrogen bonding modes during phase transition. At 318 K, the −HLOHD/ = OSR interaction peaks at 7.77 bonds while −HLOHD/−OSR slightly decreases to 7.001, demonstrating that double‐bond‐oxygen‐dominated interactions drive optimal molecular packing for thermodynamic stabilization. The crystallization process involves cooperative interactions where −HLOHD/ = OSR serves as the primary driver (Figure 3b), −HLOHD/−OSR provides complementary stabilization (Figure 3c), and both acceptor types (−OSR and = OSR) participate synergistically (Figure 3d). This cooperative hydrogen bonding network ensures the orderly microstructural transformation and solid‐state stability of the SL46 system.

### 2.3 Shape Stabilization Mechanism of SAT/LA Eutectic System

The RDFs in Figure 2i and j reveal stronger intermolecular interactions between LA and SAT compared to those between LA molecules. Furthermore, binding energy analysis demonstrates that LA/H_2_O interactions in LA/SAT are energetically more favorable than H_2_O/H_2_O interactions during both heating and cooling processes (Figure 3e,f), which indicates that heteromolecular attraction between LA and H_2_O dominates over homo‐molecular interactions between water molecules. This preferential interaction promotes the formation of a stable and dense LA/H_2_O hydrogen‐bonding network within the SL46 system, effectively preventing the segregation of H_2_O molecules into isolated clusters. The hydrogen‐bonding network enhances interfacial synergy between SAT and LA, thus improving the microstructural stability of the eutectic system.^[^ ^55^ ^]^ Molecular electrostatic potential (MEP) and charge density analyses provide further insight into these interactions (Figure 3g,h). In LA/SAT interactions, the hydroxyl hydrogen of LA carries a substantial positive charge (+0.358) and the carboxyl oxygens of SAT carry substantial negative charges (−0.678 and −0.580), enabling strong hydrogen bond formation (Figure 3g). Concurrently, negatively charged oxygens in SAT electrostatically attract positively charged regions of LA. The hydrogen bonding and electrostatic interactions significantly enhance the structural stability of the SL46 system. Figure 3h shows that the hydroxyl hydrogen of LA (+0.295) and oxygen of water (−0.516) maintain sufficient electrostatic attraction to form an effective hydrogen‐bonding network of LA−H_2_O. Although weaker than LA/SAT interactions, H_2_O can reinforce a robust LA–H_2_O–SAT network, consistent with the binding energy analysis. Collectively, these enhanced intermolecular interactions between LA and SAT components play a pivotal role in stabilizing the shape and microstructure of the LA/SAT eutectic system.

Figure 3i shows the thermal behavior of LA and SAT/LA eutectic systems during heating. Pure LA transitions from solid to liquid as the temperature exceeds its *T*_m_, exhibiting characteristic flow behavior. Similar phase‐change‐induced leakage is observed for the SL28, SL37, and SL55 eutectic systems. In striking contrast, SL46 maintains excellent shape stability throughout the heating process. This distinctive behavior correlates with the binding energy analysis presented in Figure 2d. The higher binding energy of SL46 reflects stronger intermolecular interactions within this optimized SAT/LA system. Molecular dynamics simulations reveal two key stabilizing mechanisms: 1) Enhanced heteromolecular interactions between LA and SAT compared to LA/LA interactions (Figure 2i,j), effectively preventing LA leakage. 2) Superior LA/H_2_O interactions over H_2_O/H_2_O interactions in SAT (Figure 3e,f), inhibiting phase separation of SAT components. Control experiments confirm that pure SAT undergoes water separation during heating, highlighting the critical role of optimized LA/SAT ratios. The excellent agreement between simulation and experiment validates the model's predictive power, which establishes a molecular‐level design principle for developing shape‐stable PCMs through interfacial interaction engineering.

### 2.4 Chemical Composition and Thermal Properties of SAT/LA Eutectic System

**Figure 4:**
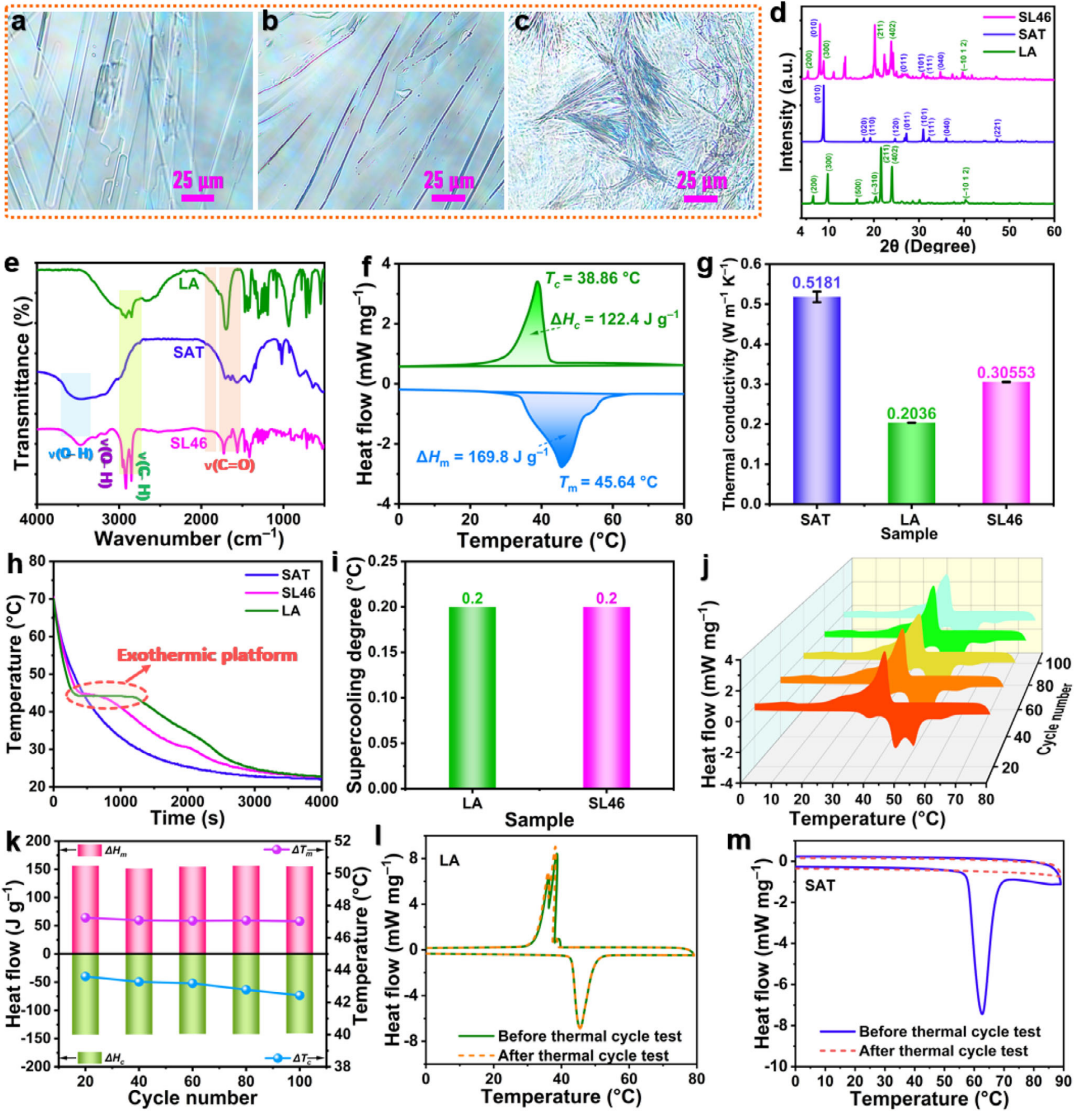
POM images of a) SAT, b) LA, and c) SL46. d) XRD patterns and e) FTIR spectra of SAT, LA, and SL46. f) DSC thermogram of SL46. g) Thermal conductivities, h) step‐cooling curves, and i) supercooling degrees of SAT, LA, and SL46. j) DSC thermograms over 100 heating/cooling cycles and k) the corresponding phase‐change enthalpy and temperature variations. DSC thermograms of l) LA and m) SAT before and after thermal cycling test.

Building on the demonstrated shape stability of the SL46 eutectic system, we further characterized its chemical composition, thermal properties, and potential application as a solvent in organic thermochromic composites. Polarized optical microscopy (POM) images of SL46, along with pure SAT and LA controls, reveal distinct crystallization patterns (**Figure** **4**a–c). Pure SAT and LA exhibit uniform, densely packed crystal morphologies with regular, elongated strip‐like shapes (Figure 4a,b), consistent with their theoretical crystal forms. In contrast, SL46 shows an interwoven growth pattern, forming needle‐like and lath‐like crystals due to synergistic interactions between SAT and LA (Figure 4c). The XRD pattern of SL46 retains characteristic peaks of both SAT and LA (Figure 4d), confirming the preservation of their individual chemical structures in the eutectic system. Notably, slight shifts in peak positions (e.g., 2θ = 8.06°, 8.90°, 20.22°, 23.88°, 27.18°, and 30.96°) and the emergence of new peaks (e.g., 2θ = 11.12°, 13.66°, and 22.36°) are observed from the SL46 eutectic system. The appearance of shifts in existing peak positions and new peaks in the SL46 eutectic is direct evidence of a modified crystal lattice structure due to novel intermolecular interactions between SAT and LA, consistent with the formation of a unique eutectic phase.^[^ ^56^ ^]^ *Fourier*‐transform infrared (FTIR) spectrum of SL46 confirms the retention of chemical structures for both components (Figure 4e), in which SAT contributes to C═O (1701, 1640, and 1562 cm^−1^) and O−H (3469 and 3290 cm^−1^) stretching vibrations, and LA contributes to C = O and C−H (2955, 2918, and 2850 cm^−1^) vibrations. While new peaks do not appear, a careful comparison shows a noticeable broadening and a slight shift to lower wavenumbers in the O−H stretching vibration region (≈3400–3300 cm^−1^) for SL46 compared to the pure components. This is a classic signature of strengthened and heterogeneous hydrogen bonding, which aligns with the MD predictions. Variable‐temperature FTIR spectroscopy further confirmed the dynamic nature of these hydrogen bonds, showing reversible changes in the O−H and C═O stretching regions during the phase transition (Figure S7, Supporting Information). The absence of significant peak shifts or new functional groups underscores that the eutectic interaction primarily alters physical packing rather than chemical bonding.

DSC analysis of SL46 reveals a Δ*H*_m_ of 169.8 J g^−1^ and crystallization enthalpy (Δ*H*_c_) of 122.4 J g^−1^ (Figure 4f), representing reductions of 5.40% and 30.29%, respectively, compared to pure LA (Figure 2b). This enthalpy decrease originates from the entropy increase upon mixing LA with SAT, which lowers the energy barrier for phase transitions.^[^ ^57^ ^]^ The *T*_m_ of SL46 (45.64 °C) shows excellent agreement (1.42% deviation) with the simulated value (45 °C) from D analysis, validating our computational model's accuracy. Despite reduced phase‐change enthalpies, SL46 exhibits enhanced thermal conductivity (0.2036 W m^−1^ K^−1^), achieving a 50.06% improvement over pure LA (Figure 4g). This enhancement stems from the superior thermal conductivity of SAT (0.5181 W m^−1^ K^−1^), which facilitates more efficient heat transfer in the eutectic system. Step‐cooling curves demonstrate distinct temperature plateaus for both LA and SL46 (Figure 4h), corresponding to latent heat release during crystallization. Notably, SL46's plateau is narrower due to its lower Δ*H*_c_ and higher thermal conductivity. In contrast, SAT shows no crystallization plateau, indicating severe supercooling. SL46 maintains minimal supercooling (0.2 °C, Figure 4i). Comparable to pure LA, such a low supercooling level is particularly advantageous for reversible thermochromic applications requiring precise temperature responses.

To evaluate the thermal reliability of the SL46 eutectic system, we performed 100 heating/cooling cycles and analyzed the phase‐change characteristics. Figure 4j demonstrates that the DSC thermograms maintain consistent endothermic and exothermic profiles throughout the cycling tests. Both the phase‐change enthalpies and temperatures show minimal variation (Figure 4k), with the Δ*H*_m_ and Δ*H*_c_ at the 100th cycle measuring 155.3 and 140.9 J g^−1^, respectively. These values represent only 0.7% and 1.5% deviations from the 20th cycle measurements, demonstrating excellent phase change reversibility comparable to pure LA (Figure 4l). This confirms that SAT incorporation preserves the long‐term thermal cycling stability of the eutectic system. In contrast, SAT alone shows no detectable phase change reversibility after thermal cycling (Figure 4m). Thermogravimetric analysis (TGA) reveals distinct thermal degradation behaviors for the system components (Figure S8, Supporting Information). LA exhibits single‐step degradation between 167–282 °C. In contrast, SAT shows a three‐stage degradation process, corresponding to dehydration at 82.5 °C (38.96% mass loss), structural transition at 136.2 °C, and sodium acetate decomposition at 570.6 °C (21.42% mass loss). Notably, SL46 demonstrates enhanced thermal stability compared to pure SAT, with its initial degradation temperature elevated to 107.4 °C. This improvement stems from the stabilizing intermolecular interactions, particularly the hydrogen‐bonding network involving water molecules, between LA and SAT components. The system's thermal stability profile, combining LA's single‐step degradation with SAT's characteristic transitions, confirms the formation of a stable eutectic system suitable for low‐temperature applications. Based on the simulation and experimental results, the performance enhancement of the SL/SAT eutectic system is attributed to three key mechanisms: 1) strong LA–SAT and LA–H_2_O interactions prevent LA leakage; 2) preferential LA–H_2_O interactions over the H_2_O–H_2_O interactions inhibit SAT phase separation (water droplet formation); 3) this synergistic network maintains a homogeneous microstructure throughout cycling to prevent structural degradation. Considering that SAT dehydrates at elevated temperatures, we evaluated the phase‐change behaviors of SL46 multiple dehydration‐rehydration cycles (see Section S2, Supporting Information, for the procedure). After the first cycle, SL46 retained its latent heat capacity (Figure S9, Supporting Information), demonstrating that its crystalline water can be recovered by rehydration under high humidity. With subsequent cycles, the latent heat decreased slightly before stabilizing, indicating an irreversible dehydration component in SAT that cannot be restored by simple humidification. Despite this, SL46 exhibits a satisfactory latent heat capacity (Δ*H*_m_) of 105.8 J g^−1^.

### 2.5 Thermochromic Performance and Mechanism

After successfully fabricating leakage‐free solid–liquid PCMs, we prepared two types of thermochromic composites using CVL and DEABPF as chromogenic agents, BPA as the color developer, and SL46 as the solvent. These two composites, designated as B@TC (CVL‐based) and P@TC (DEABPF‐based), exhibit dynamic coloration and decoloration through intermolecular electron transfer. Their fast color development, excellent oil solubility, and distinct color transitions make them highly suitable for thermochromic applications.^[^ ^58^, ^59^ ^]^ The chemical compositions of B@TC and P@TC were confirmed by energy‐dispersive X‐ray spectroscopy (EDX), FTIR spectroscopy, and XRD. EDX spectra reveal the presence of C, O, N, and Na, with uniform elemental distribution (Figure S10, Supporting Information), indicating the incorporation of CVL, DEABPF, and SAT. FTIR spectra display characteristic bands of BPA (phenolic hydroxyl at 1611 and 3353 cm^−1^) and CVL (C═O: 1742 cm^−1^; C−O−C: 1076 and 1192 cm^−1^) as shown in Figure S11a (Supporting Information). However, after mixing CVL and BPA with SL46, the C═O band at 1742 cm^−1^ disappears in B@TC, suggesting a ring‐opening reaction between CVL and BPA. Similarly, the C═O peak of DEABPF is absent in P@TC due to its reaction with BPA (Figure S11b, Supporting Information). XRD analysis confirms the presence of SL46 in both composites (Figure S12, Supporting Information), while the diffraction peaks of CVL, DEABPF, and BPA are negligible, likely due to their low concentrations in B@TC and P@TC.

**Figure 5:**
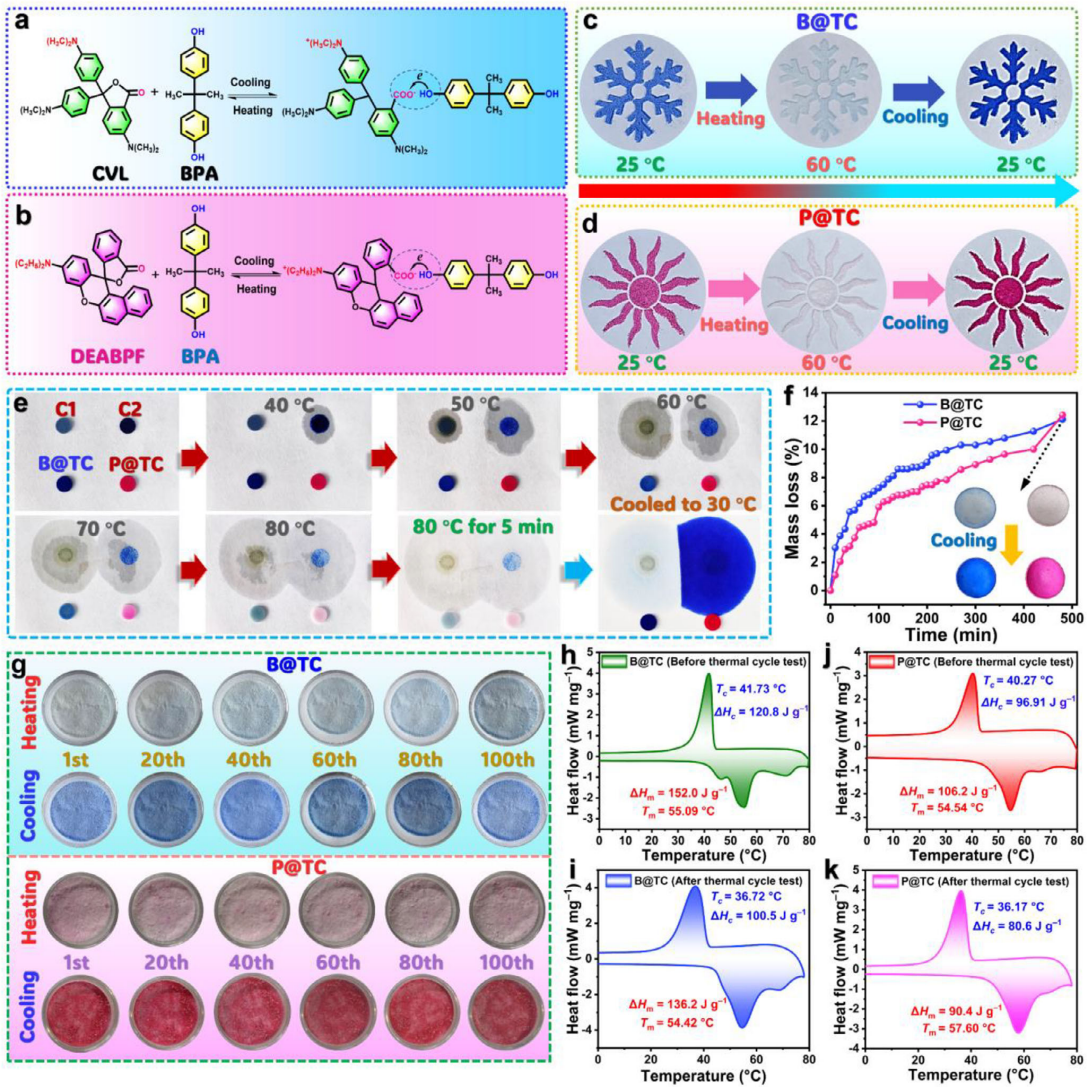
Proposed color change mechanisms of a) CVL‐based and b) DEABPF‐based thermochromic materials. Thermochromic behavior of c) B@TC and d) P@TC during heating and cooling cycles. e) Temperature‐dependent optical images of C1, C2, B@TC, and P@TC. f) Leakage rates of B@TC and P@TC 80 °C (inset: photographs of samples after isothermal treatment and subsequent cooling to room temperature). g) Cyclic thermochromic stability of B@TC and P@TC under repeated thermal cycling. DSC analysis of B@TC h) before and i) after thermal cycling, and P@TC j) before and k) after thermal cycling.

As shown in **Figure** **5**a,b, the open‐ring structures of CVL and DEABPF are inherently unstable and exhibit a strong tendency to revert to their closed‐ring forms. When the temperature drops below the *T*_m_ of SL46, CVL and DEABPF act as electron donors, releasing electrons that are captured by BPA (the electron acceptor). This electron transfer induces a structural rearrangement in CVL and DEABPF, leading to the opening of their lactone rings and the formation of ionic compounds with BPA. This process is further confirmed by FTIR spectroscopy (Figure S11, Supporting Information). In the solid state, SL46 suppresses molecular thermal motion, restricting the mobility of CVL and DEABPF chain segments. This hinders the reversion of the open‐ring forms to their closed‐ring states, causing B@TC and P@TC to appear blue and pink, respectively (Figure 5c,d). Upon heating above *T*_m_, SL46 transitions to a liquid state, weakening the interaction between BPA and the chromophores. BPA releases electrons, while CVL and DEABPF recover their closed‐ring configurations. The resulting dissociation of the ionic compounds leads to a color transition from blue to colorless for B@TC and from pink to colorless for P@TC. Through repeated temperature cycling, B@TC and P@TC demonstrate fully reversible thermochromism, driven by dynamic intermolecular electron transfer.

The shape stability of B@TC and P@TC was evaluated by heating the samples on a hot plate while monitoring their physical appearance with a digital camera. For comparison, two conventional thermochromic materials, C1 (containing CVL, BPA, and LA) and C2 (containing CVL, BPA, and ethyl stearate), were used as controls. As shown in Figure 5e, both control samples exhibit significant leakage when heated from room temperature to 80 °C, attributable to the solid‐liquid phase transition of their solvent components. In contrast, B@TC and P@TC maintained their structural integrity without any observable leakage, demonstrating the superior shape stability imparted by the SL46 eutectic solvent system. Quantitative analysis reveals that B@TC and P@TC show only minimal mass loss rates of 12.12% and 12.42%, respectively, after prolonged heating at 80 °C (Figure 5f). This mass loss is attributed primarily to the dehydration of SAT, evolving as water vapor, and not to the leakage of the liquid PCM solvent. The solid framework itself maintains its structural integrity. TGA results indicate no mass loss for DEABPF below 340 °C (Figure S13, Supporting Information). Although BPA and CVL show mass losses of 14.65 wt.% and 4.18 wt.%, respectively, below 150 °C, their low mass fractions in the thermochromic systems render these losses negligible. The formulations B@TC and P@TC have a mass ratio of CVL (or DEABPF) to BPA to SL46 of 1:0.4:50. Consequently, during heating to 80 °C, the mass loss is dominated by the dehydration of SAT, with minimal contribution from the other components. Following cooling to room temperature, both composites fully regained their original coloration, indicating that the dehydrated SAT matrix effectively retained the thermochromic components (CVL/DEABPF and BPA) within the system. The composites exhibit excellent reversible thermochromic performance through 100 heating/cooling cycles (Figure 5g), consistently transitioning between colored (blue for B@TC, pink for P@TC) and colorless states. Furthermore, digital photographs confirmed that both composites maintained their structural integrity without any visible exudation or change in morphology after 100 heating/cooling cycles (Figure S14, Supporting Information). However, DSC analysis reveals a gradual decrease in phase‐change enthalpies after repeated cycling (Figure 5h–k). For B@TC, the Δ*H*_m_ and Δ*H*_c_ decreased from 152.0 to 136.2 J g^−1^ and from 120.8 to 100.5 J g^−1^, respectively. Similarly, P@TC showed reductions of 14.88% in Δ*H*_m_ and 16.83% in Δ*H*_c_. These changes are ascribed to SAT dehydration at elevated temperatures, as confirmed by TGA. This dehydration process does not result in the leakage of the organic solvent (LA) or thermochromic components, which remain trapped within the resulting solid matrix. Thus, the latent heat capacity is slightly diminished while maintaining the material's shape stability and functionality over repeated cycles, which is essential for reversible thermochromic applications.

To confirm the changes in the eutectic structure of SL46 after the repeated thermal cycling, the chemical structures of both B@TC and P@TC after the thermal cycling are determined by FTIR and XRD. The obtained results (Figure S15, Supporting Information) show a sharp decrease in O−H bond intensity in their FTIR spectra and the disappearance of characteristic SAT trihydrate peaks in their XRD patterns, confirming the dehydration of SAT trihydrate. A new diffraction at 2θ = 8.86° appeared in P@TC after thermal cycling, attributed to the appearance of dehydrated SAT (Figure S16, Supporting Information). Crucially, the diffraction peaks formed by the eutectic structure of SL46 (e.g., at 2θ = 11.12°, 13.66°, and 22.36°) are well maintained. This demonstrates that while dehydration alters the eutectic composition (slightly diminishing latent heat), the core eutectic structure, stabilized by the persistent strong intermolecular interactions between LA and the SAT matrix, remains intact. This preserved structure is why the material retains its excellent shape‐stabilizing function and reversible thermochromism even after repeated thermal cycling.

### 2.6 Temperature Indication Performance

**Figure 6:**
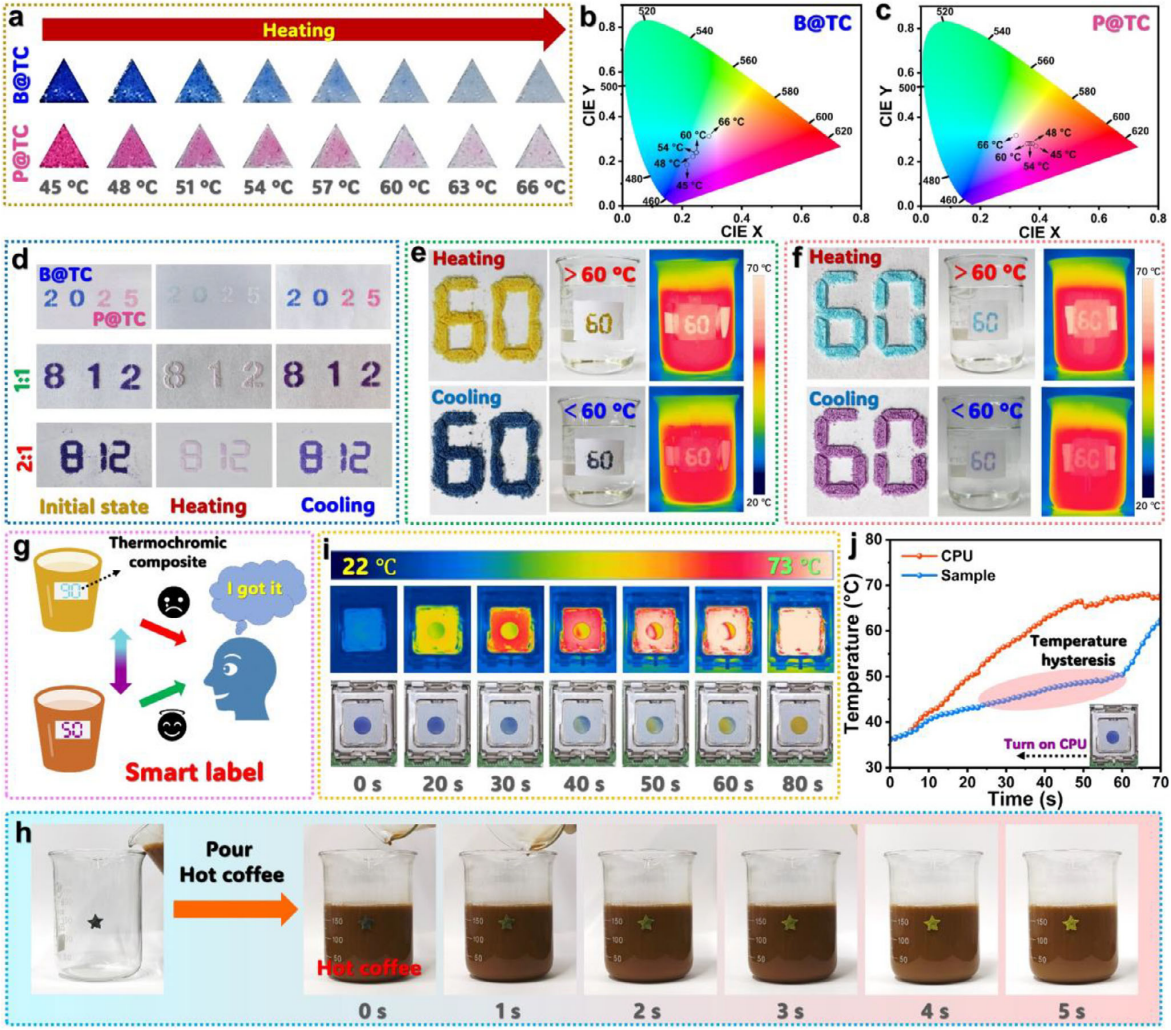
a) Digital photographs showing temperature‐dependent color evolution of triangular B@TC and P@TC samples. CIE chromaticity diagrams showing the color coordinates of b) B@TC and c) P@TC at increasing temperatures. d) Thermochromic behavior of numerical displays fabricated with different CVL/DEABPF mass ratios. Combined digital and infrared thermographic images showing the thermochromic behavior of e) methyl orange‐doped B@TC and f) methylene blue‐doped P@TC during thermal cycling. g) Conceptual illustration of thermochromic composites as temperature indicators. h) Practical demonstration of a pentagram‐shaped B@TC/methyl orange label responding to hot coffee temperature. i) Infrared and visual images, and j) corresponding temperature profiles of a CPU with an integrated B@TC/methyl orange sensor for thermal monitoring application.

The developed B@TC and P@TC composites demonstrate excellent reversible thermochromic properties, positioning them as promising candidates for temperature indication, information encryption, and data storage applications. **Figure** **6**a illustrates the visual thermochromic transition of both composites across the temperature range of 45–66 °C. The composites maintain their original coloration (blue for B@TC, pink for P@TC) when the ambient temperature remains below their respective *T*_m_’s (55.09 °C for B@TC and 54.54 °C for P@TC, as determined by DSC analysis). Upon exceeding these threshold temperatures, both materials undergo gradual color fading, transitioning from their characteristic colors to colorless states. This temperature‐dependent chromatic response enables precise optical temperature indication, with each specific temperature corresponding to a distinct color state. To quantitatively characterize this thermochromic behavior, we employed reflectance spectroscopy and Commission Internationale d'Eclairage (CIE) colorimetric analysis. Reflectance spectra reveal systematic changes in peak intensity at 435 nm for B@TC and 358–455 nm for P@TC as temperature increases from 45 to 66 °C (Figure S17, Supporting Information), confirming the visual observations of color fading. CIE chromaticity analysis provides further validation, with coordinate shifts from 0.216 and 0.185 (45 °C) to 0.290 and 0.313 (66 °C) for B@TC (Figure 6b) and from 0.387 and 0.270 (45 °C) to 0.321 and 0.319 (66 °C) for P@TC (Figure 6c) over the same temperature range. These quantitative measurements demonstrate excellent correlation between instrument‐detected color changes and visual observations, confirming the reliability of these composites as optical temperature indicators.

In addition to the color transitions from blue and pink to colorless, the developed B@TC and P@TC composites exhibit multiple reversible color changes, broadening their potential applications. As shown in Figure 6d, the molded numbers made from B@TC and P@TC demonstrate reversible thermochromic behavior, transitioning from blue to colorless and pink to colorless, respectively, during heating and cooling cycles. When the mass ratio of CVL to DEABPF is set at 1:1, the thermochromic composite shifts from purple to light upon heating. Similarly, a 2:1 mass ratio of CVL/DEABPF results in a color change from dark blue to light pink. Beyond blending CVL and DEABPF, the color of B@TC and P@TC composites can be further tuned by incorporating dyes. For example, adding methyl orange to B@TC yields a dark blue color below 60 °C and golden above 60 °C (Figure 6e). Incorporating methylene blue into P@TC produces purple below 60 °C and turquoise above 60 °C (Figure 6f). These distinct color transitions enable the composites to provide immediate visual warnings within specific temperature ranges, enhancing their usability and functionality. As illustrated in Figures 6e,f, when the water temperature in a beaker exceeds 60 °C, the number label undergoes a noticeable color change, alerting users to avoid scalding (Figure 6g). This confirms the composites' suitability for temperature indication. Figure 6h demonstrates the real‐time temperature response of B@TC with methyl orange when used as a self‐adhesive label on a beaker. Upon filling the beaker with hot coffee (> 90 °C), the label rapidly changes from dark blue to golden within 5 s, indicating its ability to provide instant thermal feedback for safe drinking.

According to the DSC thermograms (Figure 5h,j), B@TC and P@TC demonstrate a significant latent heat capacity for thermal energy storage, attributed to the SL46 solvent. Infrared thermographic images reveal that both composites (diameter: 13 mm; thickness: 2 mm) undergo a color transition from black to orange upon heating and reverse upon cooling (Figures S18a,b, Supporting Information), corresponding to gradual temperature changes. Notably, P@TC exhibits a faster color evolution than B@TC during heating and cooling due to its lower latent heat capacity. As shown in Figure S18c (Supporting Information), a temperature hysteresis occurs during heating, where SL46 absorbs thermal energy as latent heat to delay temperature rise. This effect is more pronounced in B@TC due to its higher latent heat capacity. Consequently, after heating at 80 °C for 245 s, P@TC reaches a maximum temperature of 68.17 °C, exceeding that of B@TC (62.97 °C). The stored thermal energy is subsequently released through SL46 crystallization during cooling. By leveraging SL46's latent heat storage and release, the developed B@TC and P@TC composites not only exhibit superior thermochromic performance but also demonstrate excellent thermal shock resistance. To illustrate this, Figure 6i presents infrared thermographic and digital images of a B@TC/methyl orange sample (diameter: 13 mm; thickness: 2 mm) placed on a computer central processing unit (CPU) in a running computer. Within 70 s, the sample maintains a lower average temperature than the CPU, confirming its thermal buffering effect (Figure 6j). Additionally, the sample's color change over time, observed in digital images, further indicates that SL46 effectively mitigates short‐term heat impact. These findings demonstrate that the developed thermochromic composites are promising for high‐temperature warning systems while providing robust thermal shock resistance.

### 2.7 Information Encryption and Storage

**Figure 7:**
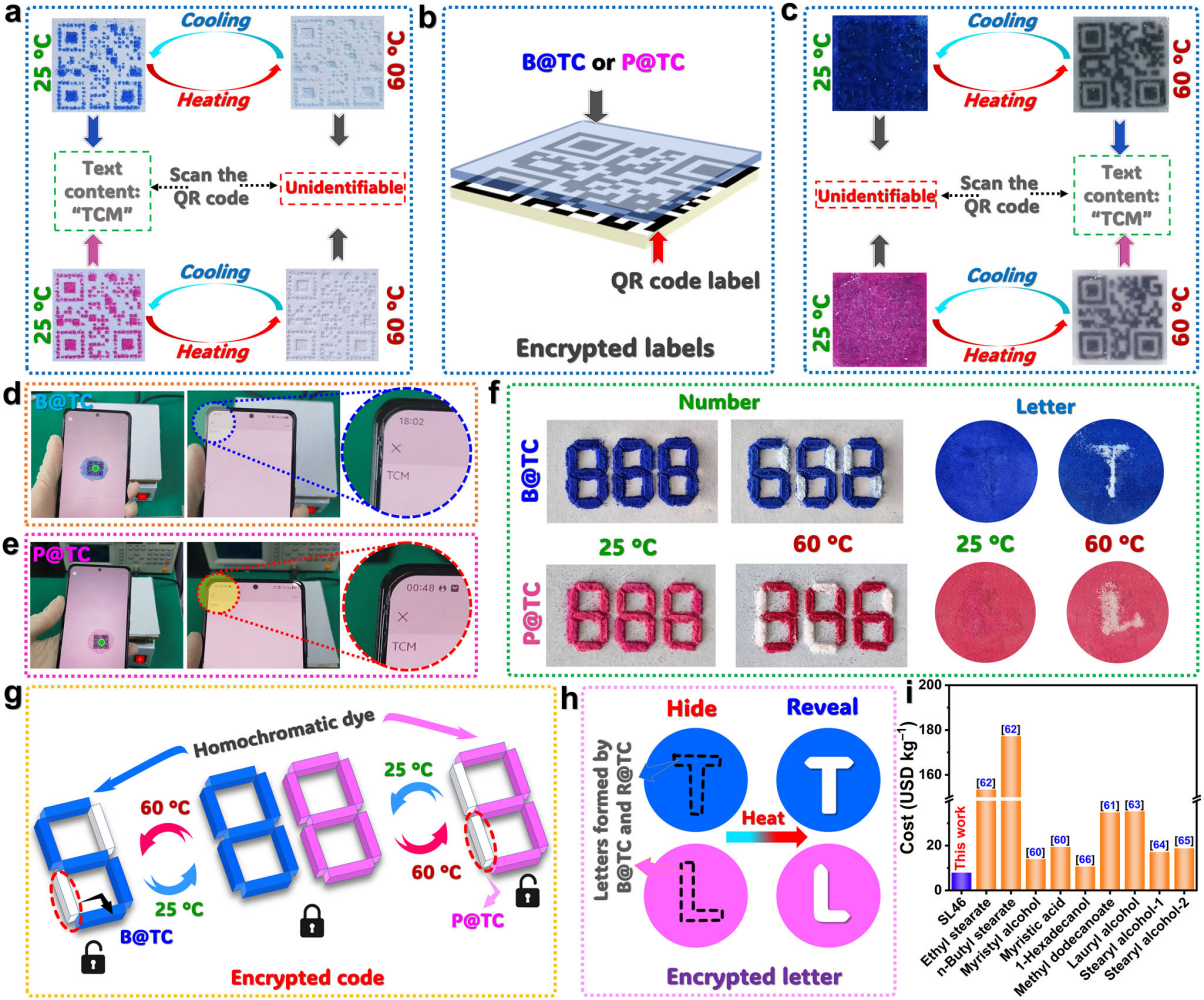
a) Digital photographs of QR codes fabricated using B@TC and P@TC, showing their temperature‐dependent visibility. b) Schematic illustration of the structure of the encrypted QR code label. c) Digital photographs showing the temperature‐triggered encryption/decryption of QR code labels based on B@TC and P@TC. Successful smartphone scanning of decrypted QR code labels at 60 °C using d) B@TC and e) P@TC, displaying stored “TCM” information. f) Digital photographs showing thermal‐triggered encryption and decryption of alphanumeric information (numbers and letters) using B@TC and P@TC. g,h) Schematic illustration depicting information encryption/storage mechanisms enabled by B@TC and P@TC. i) Cost comparison of solvents (SL46 vs CVL‐based solvents) used in thermochromic composites, highlighting the economic advantage of SL46.

Beyond temperature indication, we demonstrate the potential of SL46‐based thermochromic composites (B@TC and P@TC) for information encryption and storage. As illustrated in Figure S19 (Supporting Information), quick response (QR) codes generated using B@TC and P@TC were affixed to paper substrates. At low temperatures (< 60 °C), these codes are scannable by smartphones, revealing stored “TCM” information (**Figure** **7**a). Upon heating to 60 °C, the codes become colorless and unscannable, enabling applications in food safety or overheating warnings for industrial equipment. Figure 7b schematically depicts an encryption application. A thermochromic layer (B@TC or P@TC) was applied over a standard QR code label. Below 60 °C, this layer obscures the underlying code, preventing scanning and information retrieval (Figure 7c). When heated above 60 °C, the thermochromic layer becomes transparent, showing the QR code for successful scanning by smartphones. This temperature‐dependent decryption ensures information protection, with access granted only under appropriate thermal conditions. Figure 7d,e confirms that heating the encrypted QR code to 60 °C renders the “TCM” information scannable, validating B@TC/P@TC as effective encryption layers for temperature‐limited access.

Figure 7f demonstrates thermal‐triggered information storage. B@TC (blue) and P@TC (pink) were used to conceal alphanumeric data. At room temperature, only decoy numbers (“888”) are visible, as the composites are in their “OFF” state. Heating to 60 °C activates (“ON” state) the composites, revealing hidden numbers (“652” for B@TC, “346” for P@TC) and letters (“T”, “L”) on circular plates (Figure 7g,h). The reversible thermochromism allows repeated hiding and revealing of information via cooling/heating cycles, enhancing information security. Critically, the SL46 solvent in these composites costs ≈7.9478 $ kg^−1^ , significantly lower than solvents used in reported CVL‐based systems (Figure 7i).^[^ ^60^, ^61^, ^62^, ^63^, ^64^, ^65^, ^66^ ^]^ With the introduction of SL46 eutectic in the thermochromic materials, the developed thermochromic systems solve a critical problem hindering conventional thermochromic systems:

(1) Leakage‐free & scalable fabrication: Conventional thermochromic systems using solid–liquid PCMs as solvents leak (as shown in our Figure 5e for controls C1 and C2). Our SL46‐based composite is completely leakage‐free, enabling robust, stand‐alone devices without containers.
(2) Dual functionality (energy + indicator): Unlike many purely thermochromic systems, our material possesses significant latent heat capacity (>150 J g^−1^). This allows it to not only indicate temperature change but also buffer against thermal shock (as demonstrated in Figure 6i and j for CPU monitoring). This dual functionality is highly desirable for real‐world applications like electronics protection.
(3) Cost‐effectiveness & simplicity: As highlighted in Figure 7i, our solvent system is very low‐cost. More importantly, it is prepared by a simple one‐pot mixing process, bypassing the complex, expensive, and lab‐scale encapsulation or porous support techniques required to stabilize conventional leaky PCMs.

As shown in Table S3 (Supporting Information), the key metrics of SL46, such as stabilization process, latent heat, cycling stability, and production cost, are superior to other PCMs used in the shape‐stable thermochromic systems as reported in literature. The cost advantage, combined with straightforward processing, underscores the strong potential for scalable, shape‐stable thermochromic applications. The operational temperature of this system is constrained by the phase transition of the SL46 eutectic (≈45–66 °C), which defines its effective thermochromic range. Although the composite is shape‐stable at higher temperatures (e.g., 80 °C), prolonged exposure above 80 °C accelerates SAT dehydration and degrades latent heat capacity. Consequently, for long‐term functionality, an upper temperature limit of 70 °C is recommended. These characteristics make the system ideal for human‐centric temperature indication, low‐grade thermal management, and secure information systems, where its cost‐effectiveness and simplicity are key advantages.

## 3 Conclusion

In conclusion, we have developed a facile eutectic strategy to fabricate scalable, leakage‐free thermochromic phase‐change composites for reversible temperature indication and information encryption. This approach overcomes the critical leakage limitations of traditional encapsulation methods for solid–liquid PCMs by integrating SAT with LA into a stable eutectic mixture (SL46), bypassing the need for complex, costly, and lab‐scale encapsulation processes. Molecular dynamics simulations reveal significantly enhanced heteromolecular interactions between SAT and LA within SL46 compared to LA alone. These strengthened intermolecular interactions impart excellent shape stability to SL46, eliminating leakage and phase separation. Furthermore, SL46 exhibits a high latent heat capacity, minimal supercooling, and highly reversible phase‐change behavior. Leveraging these advantageous properties of SL46, the developed thermochromic composites by utilizing SL46 as the solvent demonstrate exceptional shape stability, robust cyclic reversible thermochromism, and retained reasonable latent heat capacity. Capitalizing on this superior performance, we have successfully demonstrated practical applications including temperature indicators, thermally regulated QR codes, information encryption layers, and thermal‐triggered information storage/disguise systems. Collectively, these demonstrations highlight the significant potential of these composites. The integration of molecular dynamics simulation into the design process provides a promising new pathway for developing industrial‐scale, cost‐effective, shape‐stable thermochromic materials.

## 4 Experimental Section

### Materials

Ethanol (98.5 wt.%), SAT (98.0 wt.%), LA (99.0 wt.%), BPA (98.5 wt.%), CVL (98.5 wt.%), DEABPF (98.0 wt.%), and ethyl stearate (98.5 wt.%) were purchased from Shanghai Macklin Biochemical Co., Ltd., China. All chemicals were used as received without further purification.

### Preparation of SAT/LA Eutectic Mixtures

SAT/LA eutectic mixtures were prepared at mass ratios of 2:8, 3:7, 4:6, and 5:5 (SAT:LA). In a typical procedure, SAT (1.6 g) and LA (2.4 g) were combined in a beaker and manually mixed at room temperature to achieve homogeneity. The mixture was then heated to 75 °C in an oil bath and maintained at this temperature for 30 min under magnetic stirring. After 15 min of initial stirring, the mixture was held for an additional 30 min at 75 °C to ensure complete mixing. Finally, the mixture was cooled to room temperature to solidify the eutectic mixture.

### Preparation of B@TC and P@TC

Thermochromic composites, B@TC and P@TC, were prepared using SL46 as the solvent, BPA as the electron acceptor, and either CVL or DEABPF as the electron donor, respectively. The mass ratio of electron donor/electron acceptor/solvent was fixed at 1:0.4:50. In a typical procedure, SL46 (2 g) was placed in a beaker and heated to 90 °C until fully molten. CVL (0.04 g) and BPA (0.016 g) were then added to the molten SL46, and the mixture was stirred continuously at 90 °C for 1 h. The resulting mixture was cooled to room temperature to yield B@TC. P@TC was prepared identically, substituting DEABPF (0.04 g) for CVL as the electron donor.

### Characterizations and Measurements

Details for the methods of molecular simulation, structural characterization, and performance characterization are described in detail in Sections S1 and S2 (Supporting Information).

## Conflict of Interest

The authors declare no conflict of interest.

## Supporting information



Supporting Information

## Data Availability

The data that support the findings of this study are available from the corresponding author upon reasonable request.
