# Supplementary material for: A Facile Eutectic Strategy for Scalable, Leakage‐Free Thermochromic Phase‐Change Composites Enabling Smart Temperature Labels and Secure Data Encryption
Source: Adv Sci (Weinh). 2025 Nov 12;13(5):e19934. doi: 10.1002/advs.202519934 (PMC12850072; doi:10.1002/advs.202519934)
Supplement: Supplementary file 1 — Supporting Information [file ADVS-13-e19934-s001.docx]

Supporting Information

**A Facile Eutectic Strategy for Scalable, Leakage-Free Thermochromic Phase-Change Composites Enabling Smart Temperature Labels and Secure Data Encryption**

Shiliang Zhou, Qianyi Zhang, Huan Liu,^*^ Zhiqiang Qian,^*^ and Xiaodong Wang^*^

**S. Zhou, Q. Zhang, H. Liu, X. Wang**

State Key Laboratory of Organic–Inorganic Composites, Beijing University of Chemical Technology, Beijing 100029, China

E-mail: liu.huan@mail.buct.edu.cn (H. Liu); wangxd@mail.buct.edu.cn (X. Wang)

**Z. Qian**

Key Laboratory of Green and High-End Utilization of Salt Lake Resources, Qinghai Institute of Salt Lakes, Chinese Academy of Sciences, Qinghai Provincial Key Laboratory of Resources and Chemistry of Salt Lakes, Xining, Qinghai 810008, China

E-mail: qianzq@isl.ac.cn

**Section S1. Molecular dynamic simulation**

Molecular dynamic simulation is performed in Materials Studio software package to analyze the intermolecular interactions between SAT and LA by calculating the mean square displacement (MSD), interaction energy, radial distribution function (RDF), and electrostatic potential.

**S1.1 Force fields**

In the calculation process of molecular simulation, the accuracy of the simulation results is highly dependent on the applicability of force fields. To ensure the reliability of the simulation results, it is necessary to choose the appropriate force fields. There are two methods normally used for selecting the force fields. The one is to compare the simulation results under different force fields with the experimental results to confirm the appropriate force fields for molecular simulation. The other one is to adopt the specific force fields based on the reported literature and the characteristics of the simulation systems. The COMPASS force fields are adopted according to analyzing the results as reported in literature and comparing them with the simulation systems in this work.**^[^**[**^1^**](#_ENREF_1)**^]^**

**S1.2 Simulation conditions**

To build the simulation model, we first constructed the unit cell of sodium acetate trihydrate (SAT) and the molecular chain of lauric acid (LA). The molecular chains were optimized geometrically to achieve the conformation with minimum energy. The mole ratio of SAT and LA was determined as 48:49 to in accordance with their mass ratio of 4:6. Periodic boundary conditions were employed in the simulation. The specific simulation model is shown in **Figure 2c**, in which the spheres with purple color represent Na, with red color represent O, and with white color represent H, and with gray color represent C. The parameters of the simulation model are listed in **Table S1**.

To mitigate the initial structural instability, the geometry optimization, annealing treatment, and dynamic equilibrium treatment were performed before calculation. Firstly, a geometry optimization of 20,000 steps was performed using the energy minimization method based on the Smart algorithm to reduce excessively short initial interatomic distances in the simulation system. After the geometry optimization, the structure was essentially converged, so no annealing procedure was carried out. Then a 100 ps NVT simulation under constant volume and constant temperature conditions was conducted on the optimized conformation to allow sufficient relaxation of the molecular chains at 298 K. Finally, the NPT ensemble was employed to perform dynamic equilibration processes with heating and cooling between 298–358 K at 10 K intervals, with each temperature simulated for 1000 ps. During the molecular simulations, temperature was controlled using the Nosé-Hoover method, while pressure was regulated by the Berendsen coupling method. Van der Waals interactions were treated using an atom-based potential model and electrostatic interactions were calculated using the Ewald method. A time step of 1 fs was used throughout all simulations.

**S1.3 Simulation results**

**S1.3.1 density (*ρ*)**

*ρ* is a critical thermal parameter of phase change material, and it is also an important basis to verify the accuracy of the selected force field.**^[^**[**^2^**](#_ENREF_3)**^]^** In the NPT ensemble, *ρ* is calculated by the following equation:

 (S1)

where *N* is the total number of particles in the system, *M* is the molecular weight, *V* is the volume of the system, and *N*_A_ is the Avogadro's constant.

**S1.3.2 Mean square displacement (MSD) and self-diffusion coefficient (*D_α_*)**

MSD represents the position of a particle as a function of time, which can be used to determine the diffusion, transport, and binding states of atoms or molecules.**^[^**[**^3^**](#_ENREF_4)**^]^** In an equilibrium ensemble, the MSD has to be independent of time and can be achieved by averaging the position information over time. In this case, MSD can be a measure of the deviation of the position of the particle from the reference position after moving over time, which can be determined by the following equation:

$MSD\text{=}\frac{1}{t}\int_{0}^{t} [r(t)-r(0)]^{2}dt\text{=}\left\langle\left.\left|\overrightarrow{r}(t)-\overrightarrow{r}(0)\right|^{2}\right\rangle\right.$ (S2)

where *r*(*t*) and *r*(0) represent the positions of particle at time *t* and 0, respectively. The self-diffusion coefficient (*D*) reflects the intensity of atomic mobility, which determines the relationship between the position of particles and the temperature and the pressure in the system. *D* can be calculated based on the MSD of Einstein’s diffusion law:**^[^**[**^4^**](#_ENREF_5)**^]^**

$D\text{=}\frac{1}{6}\lim_{t\to\infty} \frac{d}{dt}\sum_{i=1}^{N} \left\langle\left.\left|\overrightarrow{r}(t)-\overrightarrow{r}(0)\right|^{2}\right\rangle\right.$ (S3)

*D* is widely used to describe whether a phase transition occurs in the system.**^[^**[**^5^**](#_ENREF_6)**^]^** Specifically, the phase transition temperature can be identified by fitting the *D* data. When *D* exhibits a significant change, it indicates that a phase transition has taken place within the simulation box. In such cases, a distinct inflection point appears in the plot of *D* versus temperature. Based on the equation S3, *D* is equal to one sixth of the slope of the MSD curve.

**S1.3.3 Radial distribution function (RDF)**

RDF mainly describes the packing state of atoms and the spacing between bonds. Consequently, the particle density changes with distance from reference atoms in statistical mechanics can be described by RDF. The shape and high peaks in the RDF curves illustrate that the system is in an ordered state.**^[^**[**^6^**](#_ENREF_7)**^]^** The RDF can be calculated by the following equation:

$g\text{(}r\text{)=}\frac{dN}{\rho4\pi r^{2}dr}=\frac{1}{\rho4\pi r^{2}\delta r}\times\frac{\sum_{t=1}^{T} \sum_{j=1}^{N} \Delta N(r\to r+\delta r)}{NT}$ (S4)

where *T* is the total simulation time, *δ*_r_ is the distance between atoms, and Δ*N* is the number of particles.

**S1.3.4 Hydrogen bonding**

To gain an in-depth understanding of the influence of hydrogen bonds on thermal properties of SAT/LA, this work employed a customized script to extract the detailed information from the simulation date, including the number of hydrogen bonds, hydrogen bond lengths, hydrogen bond angles, and hydrogen bond types. To enhance the reliability and accuracy of the simulation data, three specific temperatures are selected in the cooling process under the NPT ensemble, including 338, 328, and 318 K. The hydrogen bond count is statistically evaluated by analyzing every frame of the simulation trajectory at each temperature, and its average value is also calculated.

**S1.3.5 Binding energy (*E_binding_*)**

*E*_binding_ is defined as the interaction energy (*E*_inter_), which can reflect the interaction between two components.**^[^**[**^7^**](#_ENREF_8)**^]^** The higher the *E*_binding_, the stronger the interactivity in the system. If *E*_binding_ is positive, the compatibility between the two components is good. The *E*_binding_ of SAT/LA mixtures with mass ratios of 2:8, 3:7, 4:6, and 5:5 can be calculated by the following equation:

$E_{\text{binding}}=-E_{\text{inter}}=E_{\text{A}}+E_{\text{B}}-E_{\text{total}}$ (S5)

where *E*_A_, and *E*_B_ are energies of A and B, respectively, and *E*_total_ represents the total energy of A and B.

**S1.3.6 Molecular electrostatic potential (MEP)**

MEP is crucial for visualizing the overall charge distribution of molecules and evaluating intermolecular interactions.**^[^**[**^6^**](#_ENREF_7)**^]^** During the simulation process, the initial simulation models are constructed for LA molecules with sodium acetate and H_2_O, respectively (two sets in total). Subsequently, the initial models undergo geometry optimization to obtain the accurate wavefunctions. The generalized gradient approximation (GGA) and BLYP functional are used to deal with the wave function. There exists a close relationship between MEP and electron cloud distribution, enabling the estimation and characterization of electrophilic reaction sites in terms of their size, nucleophilicity, charge density, and shape. Additionally, core electrons are treated using the DFT semi-core pseudopotential method. To accelerate the computational process, the smearing algorithm is utilized during optimization. According to the converged geometric structures, the electron density and electrostatic potential distributions are calculated for both sets of simulation models.

**Section S2. Characterizations and measurements**

**S2.1 Characterizations**

The phase-change behaviors of SAT, LA, SAT/LA eutectic mixtures, and thermochromic composites were performed by a differential scanning calorimeter (DSC, Q20, TA Instruments, USA). The thermal cycling stability of SAT, LA, SL46 (SAT/LA eutectic mixture at a mass ratio of 4:6), and SL46-based thermochromic composites was also characterized by the DSC measurement. The shape stability of SAT, LA, SAT/LA eutectics, and SL46-based thermochromic composites placed on a hot plate (JF-956A, Chang'an Jinfeng, China) at different temperatures was recorded by a smart phone. The optical microscopic images of SAT, LA, and SL46 eutectic were recorded by a polarizing microscopy (POM, XPN-300E, Shanghai Changfang Optical Instrument, China). The *X*-ray diffraction (XRD) patterns were characterized by an *X*-ray diffractometer (D/max 2500, Rigaku, Japan) under Cu Kα radiation. The chemical structure was characterized by *Fourier*-transform infrared (FTIR) spectrometer (NicoletTM iS5, USA) in the wavelength range from 400 to 4000 cm^‒1^. The variable-temperature FTIR spectroscopy (Bruker INVENIO-S.Harrick, Germany) was performed to evaluate the dynamic nature of hydrogen bonds in SL46 at a heating rate of 5 °C min^‒1^. The surface elemental composition and mapping images of SL46-based thermochromic composites were characterized by an energy-dispersive X-ray spectrometer (EDX, Oxford INCAX-Act, England). The thermal conductivity of SAT, LA, and SL46 was characterized by a HyperFlash thermal conductivity meter (NETZSCH, LFA 467, Germany). The step-cooling curves of SAT, LA, and SL46 eutectic were recorded by a *k*-type thermocouple. The thermal degradation behaviors of SAT, LA, SL46, CVL, DEABPF, and BPA were characterized by a thermogravimetric analyzer (TGA, Q50, TA Instruments, USA).

The reversible thermochromic performance and cyclic reversible thermochromic performance of SL46-based thermochromic composites was recorded by a digital camera. The mass loss of SL46-based thermochromic composite samples was characterized by an analytical balance. The samples were heated at 80 °C and weighted at every 10 min. The mass loss is calculated by the following equation.

$\text{Mass loss}(\%)=\frac{m_{0}-m_{\text{t}}}{m_{0}}\times100\%$ (S6)

where *m*_0_ represents the initial mass and *m*_t_ is the mass measured at a certain point in time during the heating process. The temperature management of SL46-based thermochromic composites was recorded by a thermal infrared imager (Testo 875-1i, Germany). The absorptivity of SL46-based thermochromic composites in the wavelength range of 0.3–2.5 μm were characterized using an ultraviolet-visible spectrophotometer (UV-3600 Plus, Shimadzu, Japan).

**S2.2 Dehydration-rehydration of SL46**

A cyclic dehydration-rehydration experiment was conducted to evaluate the dehydration-rehydration behavior and its impact on phase change performance of SL46. SL46 (0.25 g) was spread up in a surface dish and then the surface dish was placed on a hot plate at 90 °C for 10 min to procced dehydration. Then the surface dish was transferred in a glass cylinder along with a humidifier to maintain the relative humidity of 80%–85%. The surface dish was placed in the glass cylinder for 2 min for rehydration. The phase change behaviors of SL46 after the dehydration and rehydration processes were determined by the DSC measurement.

**S2.3 Application of composites as labels**

To demonstrate the applications of the thermochromic composites as labels for temperature indications, the composites were designed as different shapes. In a typical procedure of thermochromic QR codes, the thermochromic composite powders were filled in a QR code mold. Afterward, the mold was removed and the powders were heated at 60 °C to proceed a thermochromic process. The thermochromic QR codes were obtained after cooling to room temperature.


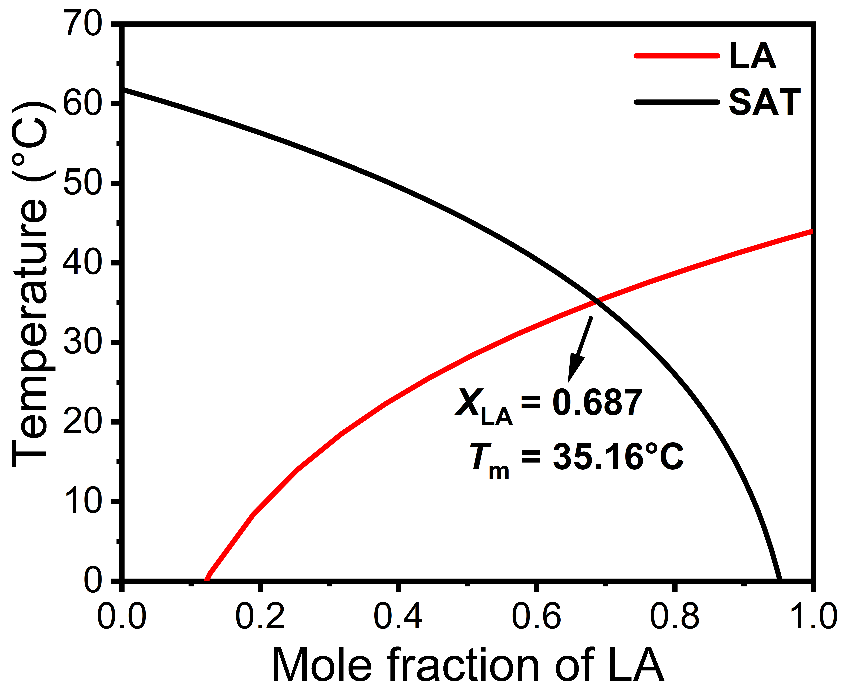


**Figure S1.** Temperature evolution of SAT/LA eutectic mixture calculated by the Schroder-Van Laar equation.

**
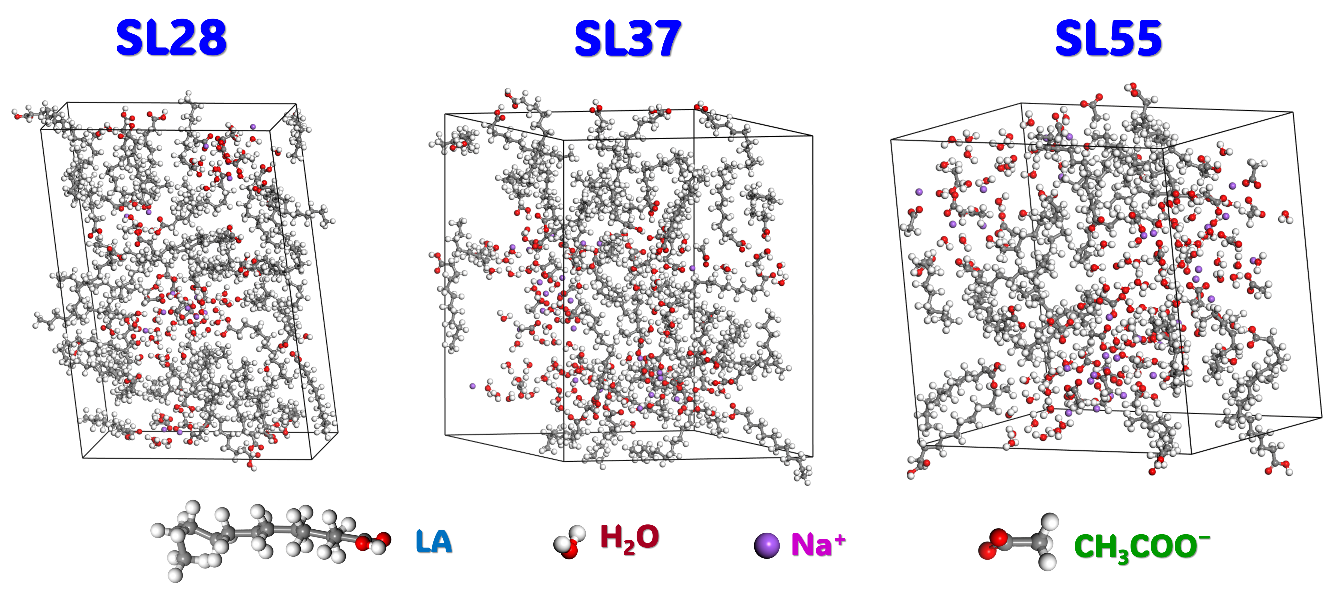
**

**Figure S2.** Snapshots of the simulation models of SL28, SL37, and SL55.

**
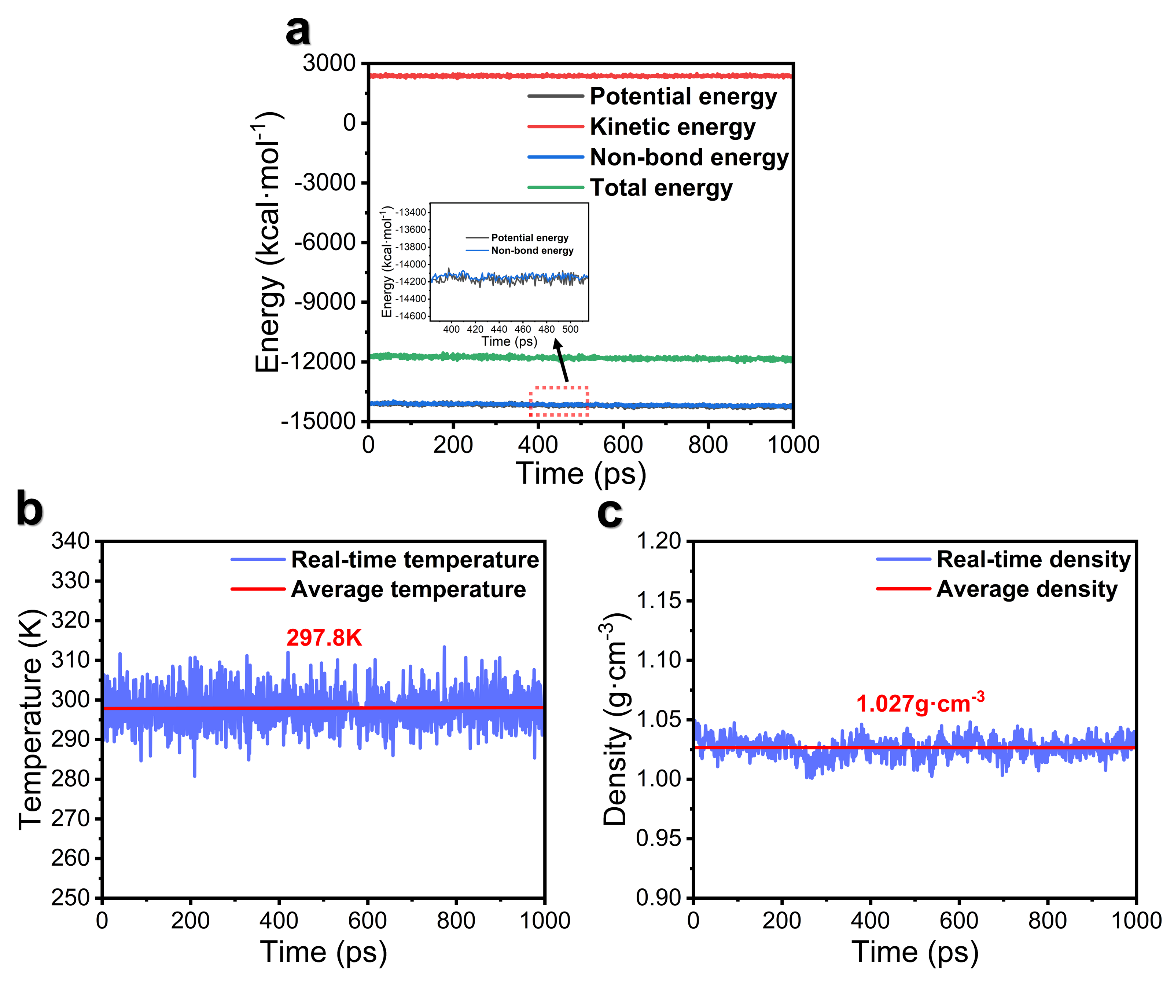
**

**Figure S3.** (a) Energy fluctuation curves (b) temperature fluctuation curve in the relaxation equilibrium process of SL46 eutectic system under NVT ensemble at 298 K. (c) Density fluctuation of SL46 eutectic system in molecular dynamics simulation at NPT ensemble at 298 K.


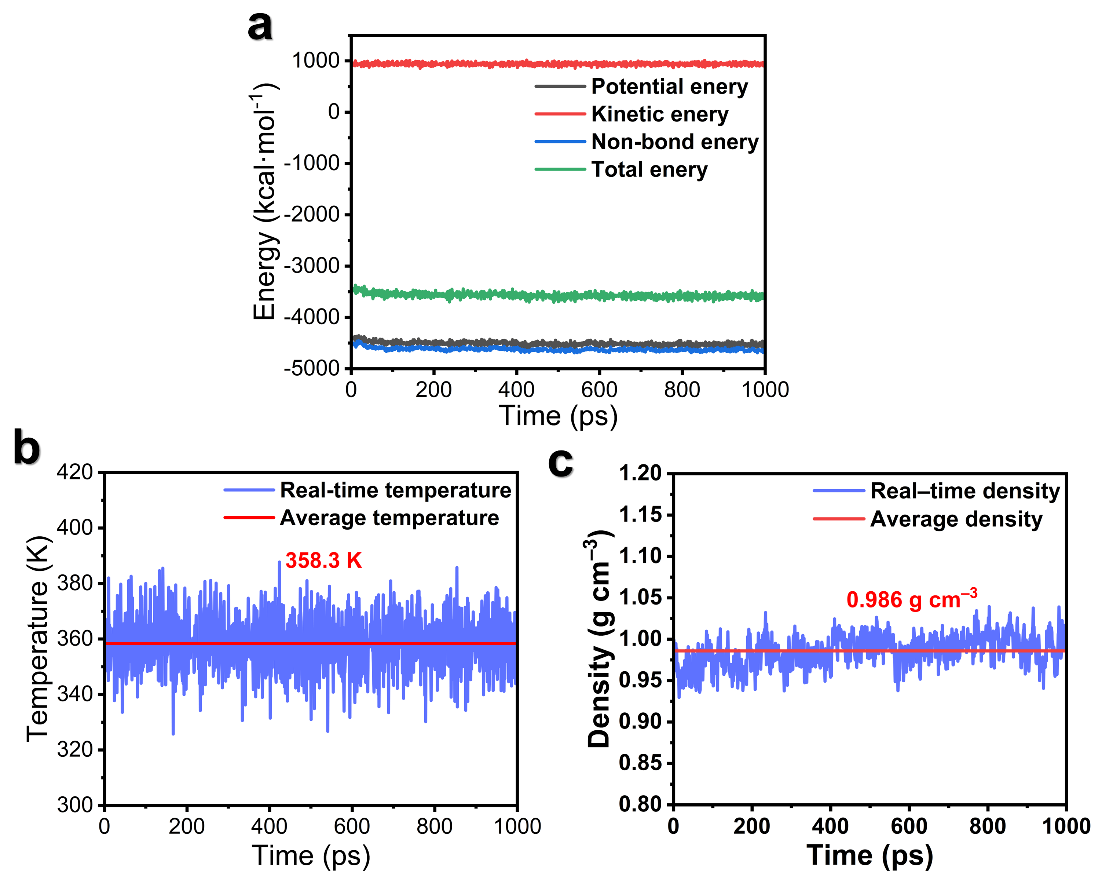


**Figure S4.** (a) Energy fluctuation curves (b) temperature fluctuation curve in the relaxation equilibrium process of SL46 eutectic system under NVT ensemble at 358 K. (c) Density fluctuation of SL46 eutectic system in molecular dynamics simulation at NPT ensemble at 358 K.


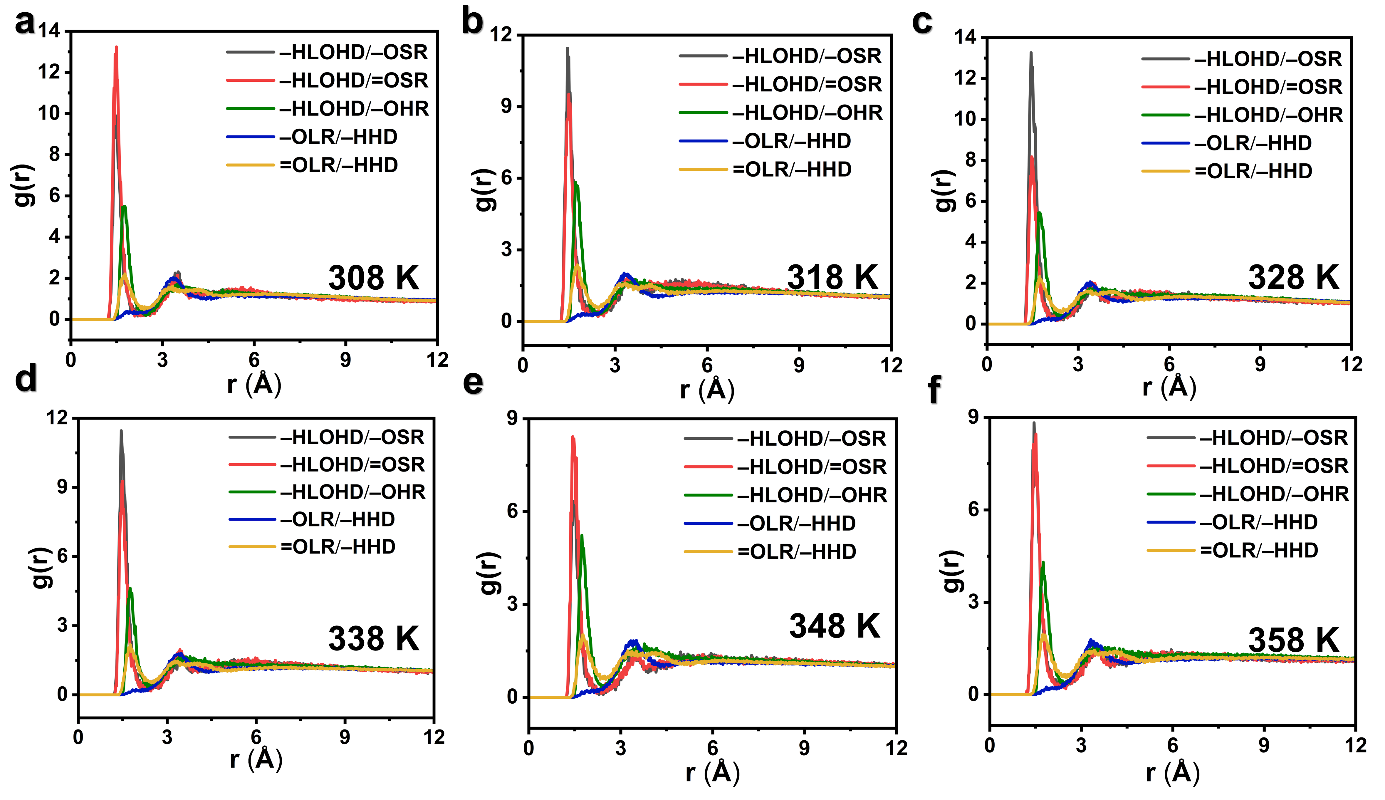


**Figure S5**. RDFs for different types of hydrogen bonding interactions at (a) 308, (b) 318, (c) 328, (d) 338 K, (e) 348 K, and (f) 358 K in the heating process.

**
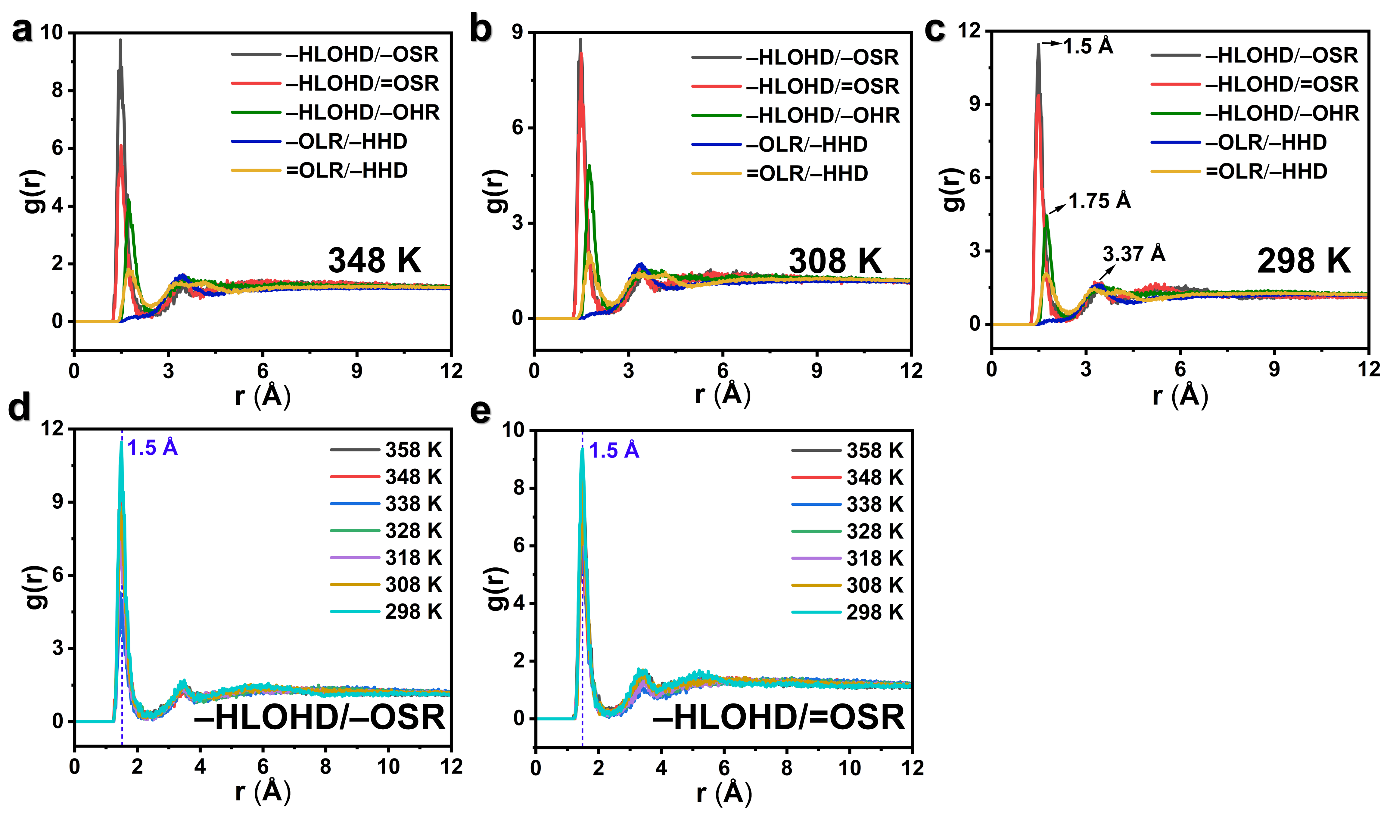
**

**Figure S6.** RDFs of different types of hydrogen bonding interactions at (a) 348, (b) 308, and (c) 298 K in the cooling process. (d) –HLOHD/–OSR and (e) –HLOHD/=OSR interactions at different cooling temperatures.

**
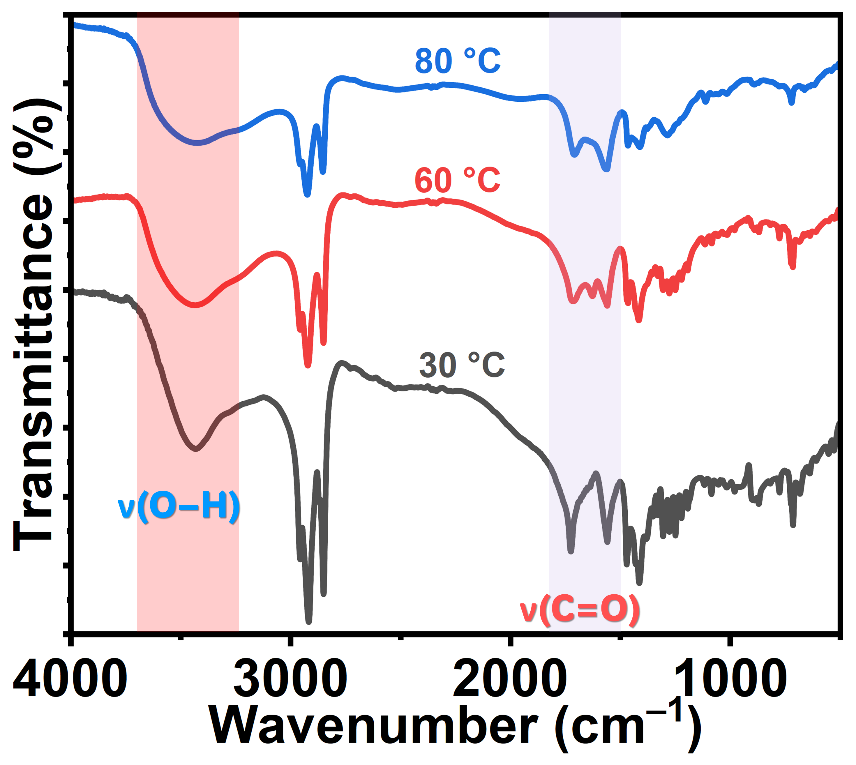
**

**Figure S7.** Variable-temperature FTIR spectra of SL46 from 30 to 80 °C.

**
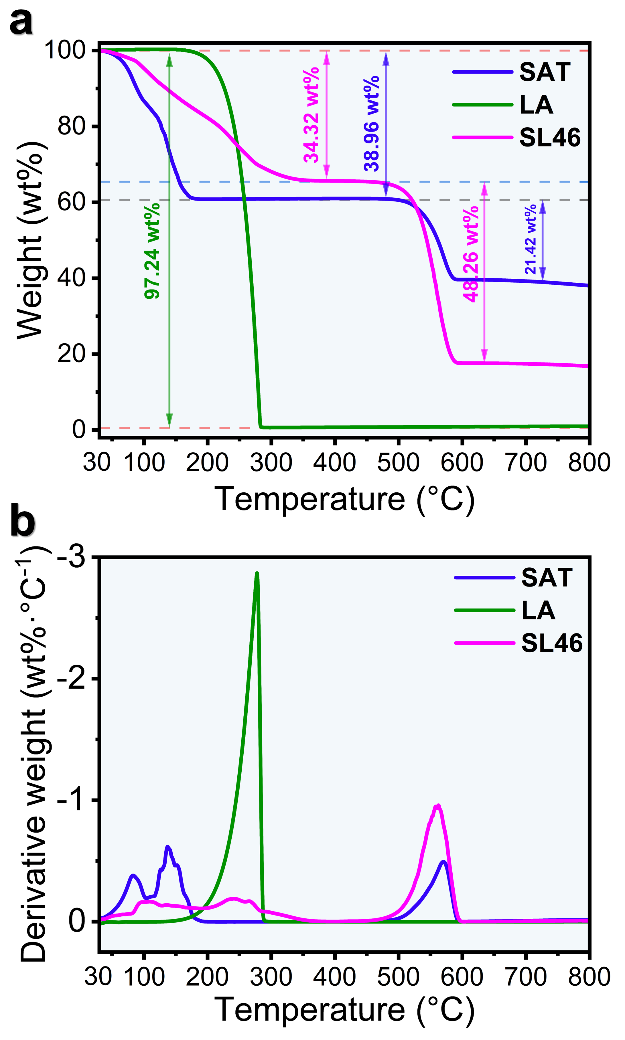
**

**Figure S8.** (a) TGA and (b) DTG thermograms of SAT, LA, and SL46.


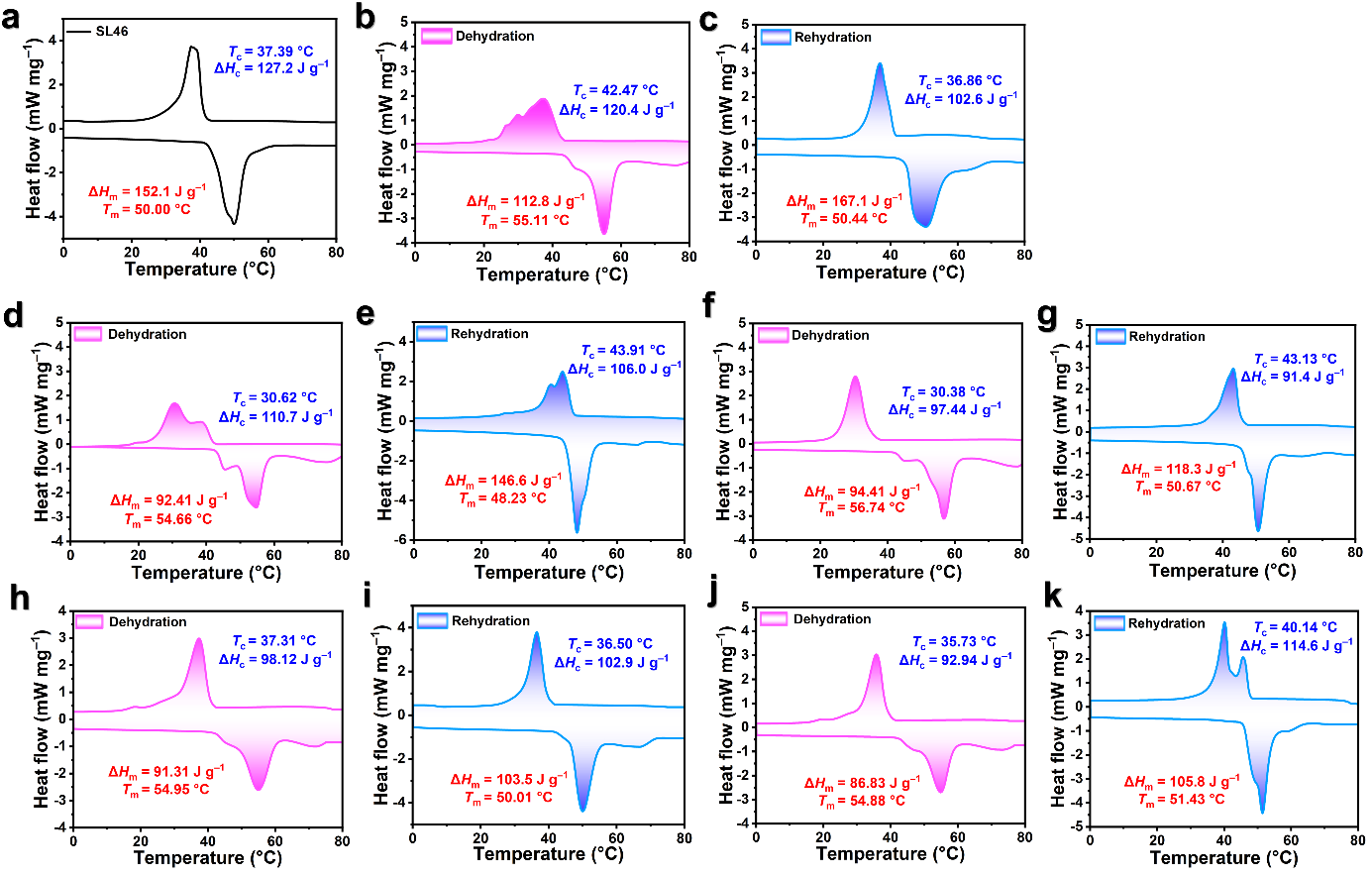

**Figure S9.** DSC thermograms of SL46 during cyclic dehydration-rehydration process: a) before dehydration-rehydration; b,c) 1st, d,e) 2nd, f,g) 3rd, h,i) 4th, and j,k) 5th dehydration-rehydration cycle.


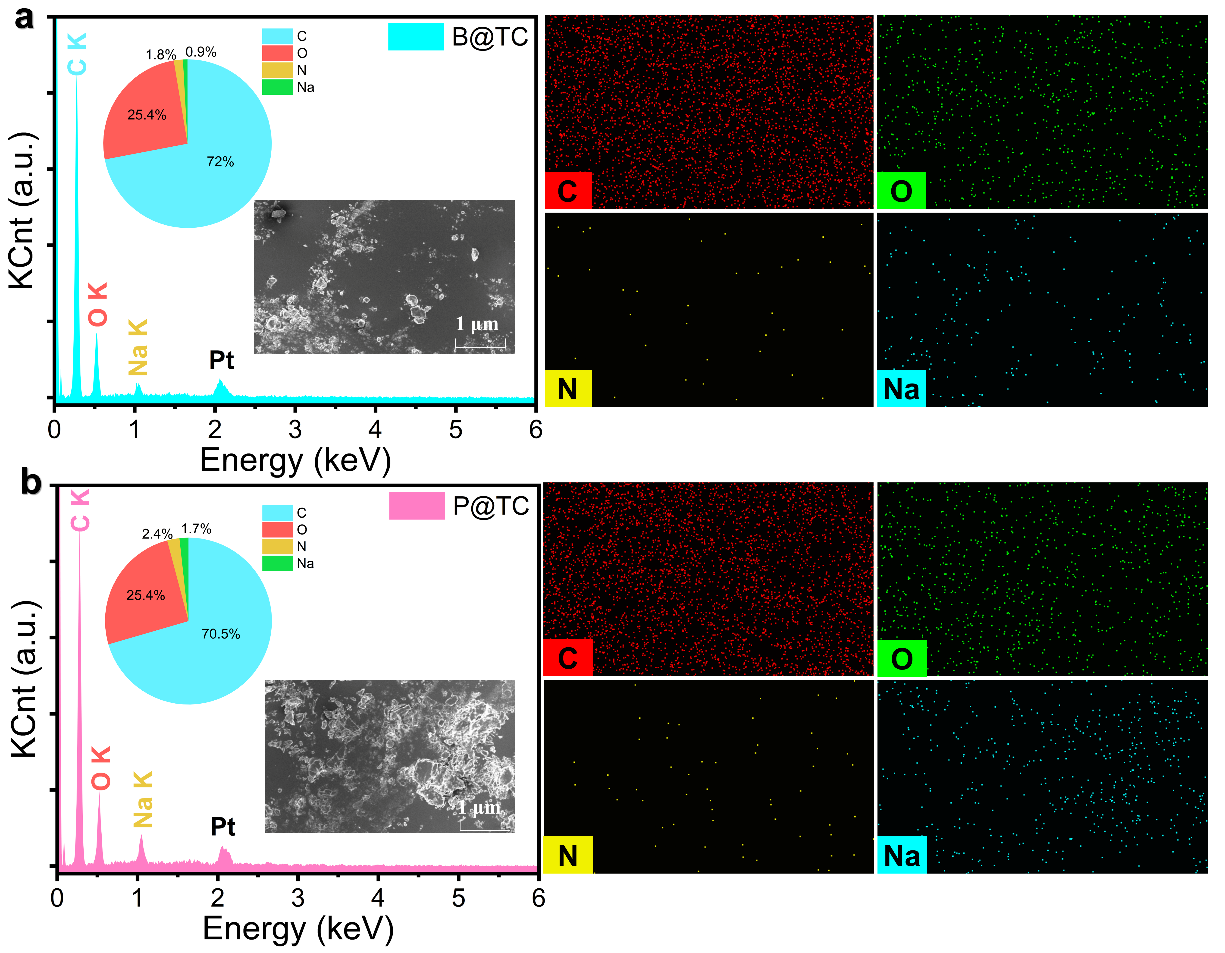

**Figure S10.** EDX patterns and the corresponding elemental mapping images of (a) CVL-based (B@TC) and (b) DEABPF-based thermochromic composites (P@TC).

**
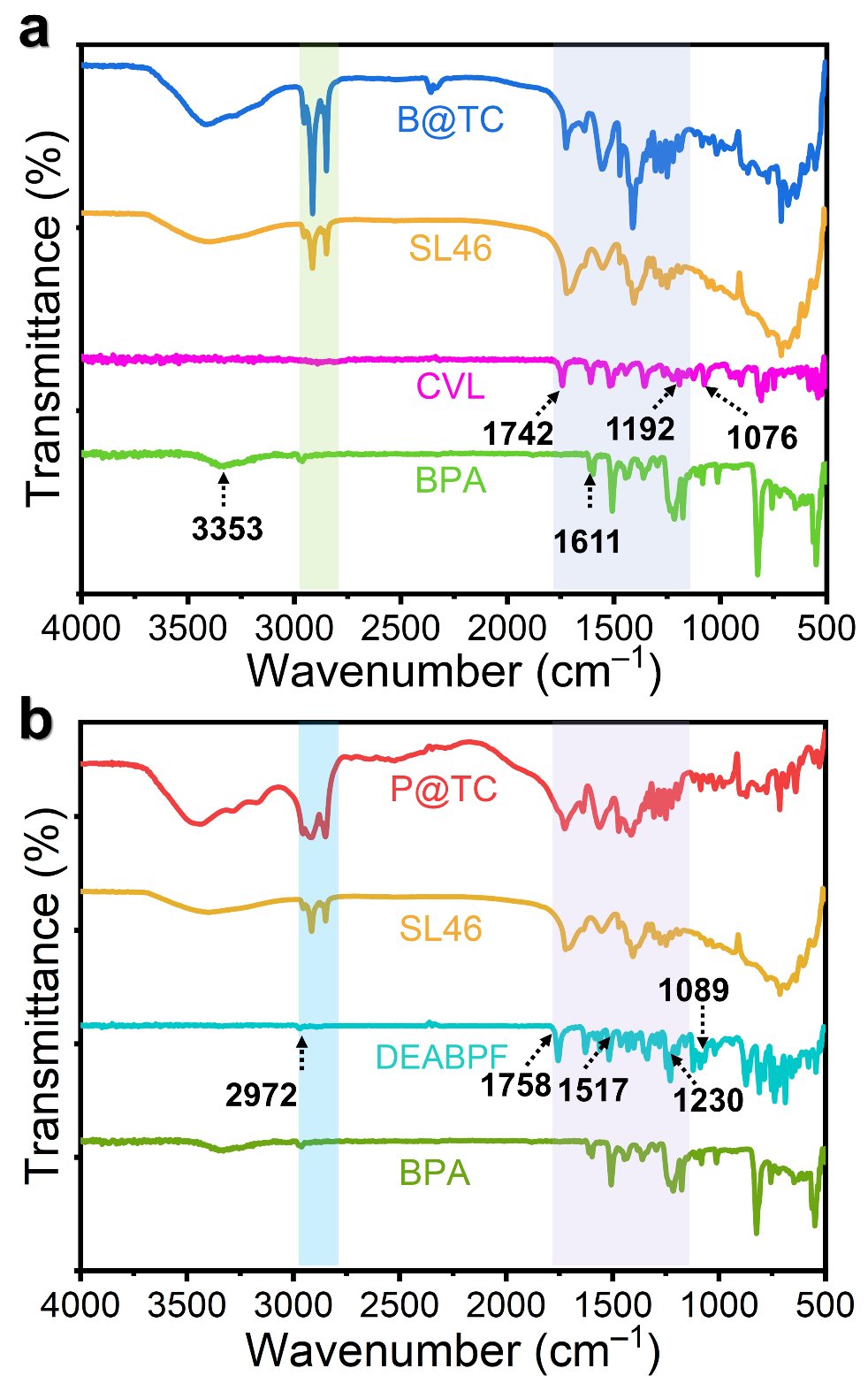
**

**Figure S11.** FTIR spectra of (a) B@TC and (b) P@TC.

**
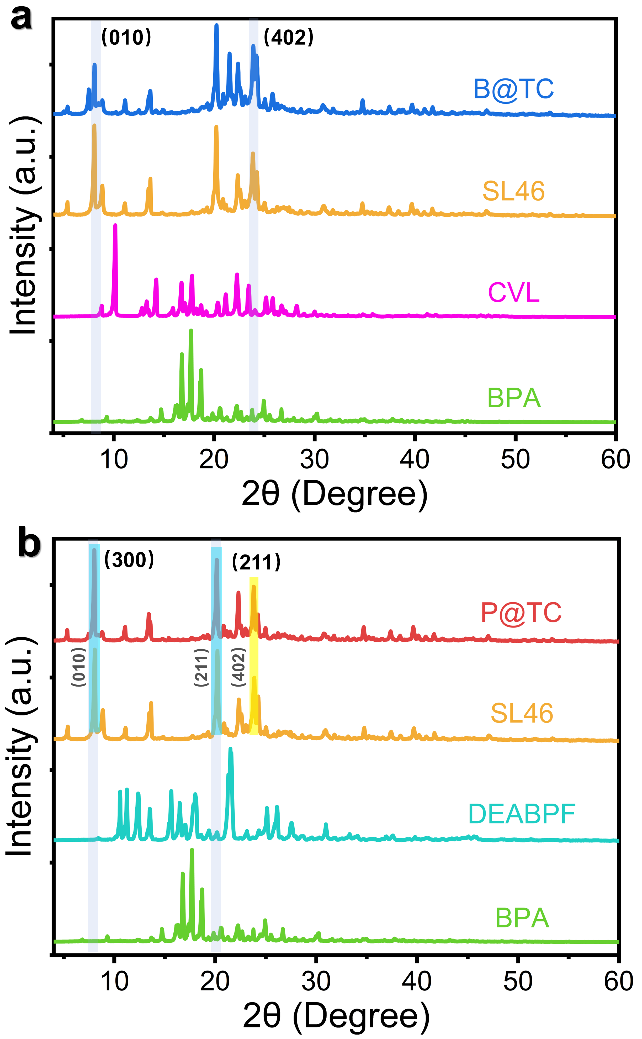
**

**Figure S12.** XRD patterns of (a) B@TC and (b) P@TC.

**
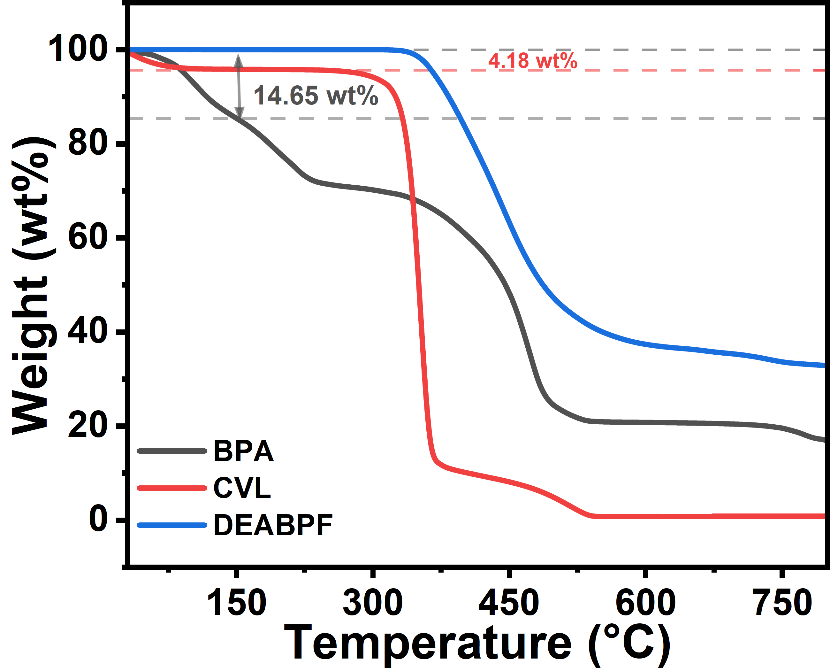
**

**Figure S13.** TGA thermograms of BPA, CVL, and DEABPF.

**
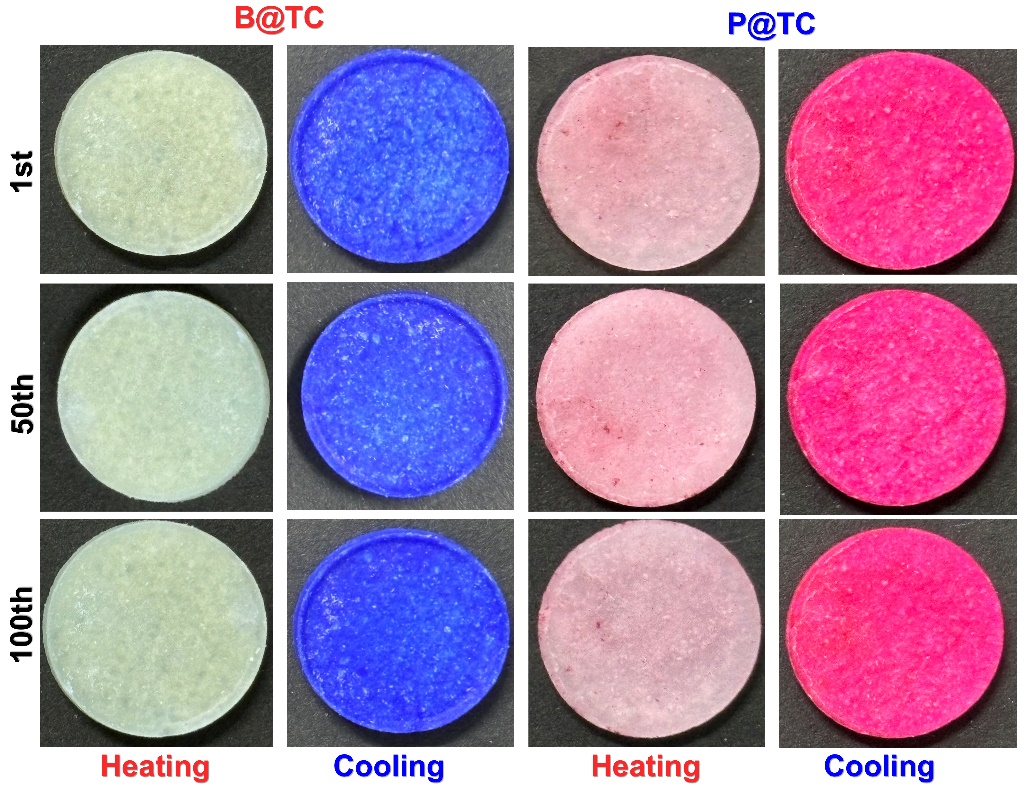
**

**Figure S14.** Optical images of B@TC and P@TC after repeated heating/cooling thermal cycling: a) 1st, b) 50th, and c) 100th thermal cycle (The B@TC and P@TC samples with a diameter of 1.3 cm were formed by a tablet press machine).

**
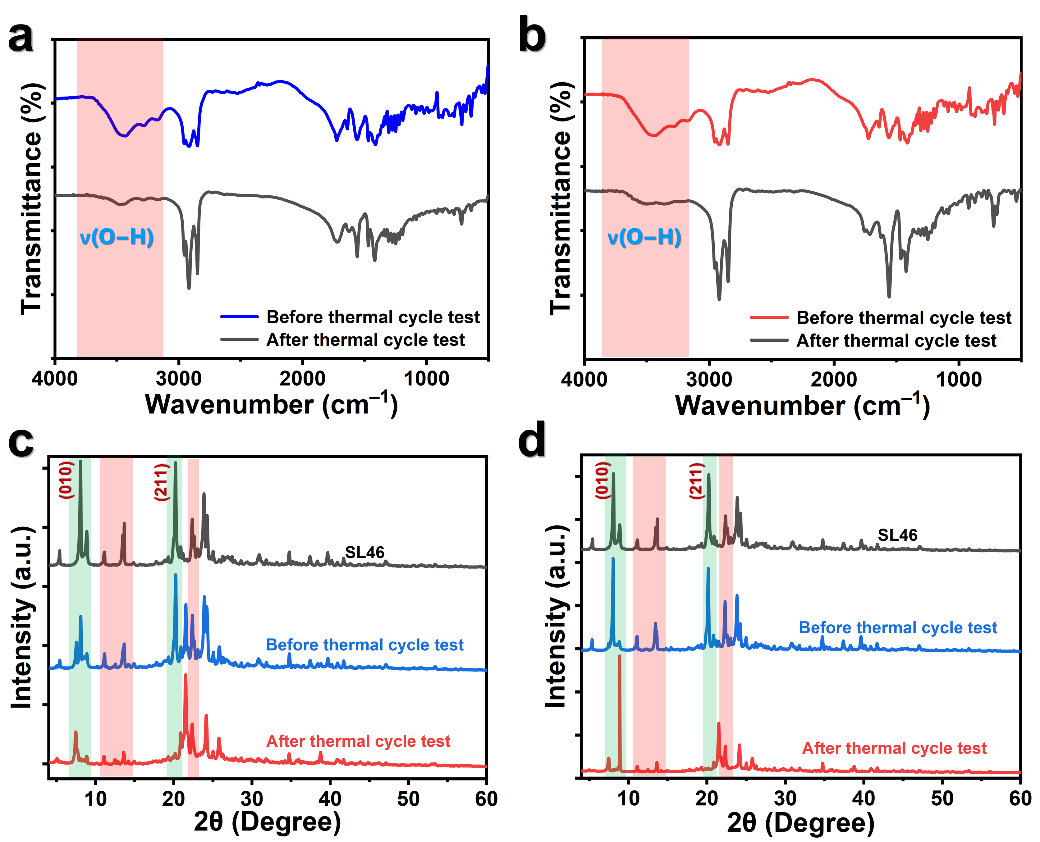
**

**Figure S15.** FTIR spectra of a) B@TC and b) P@TC before and after repeated heating/cooling thermal cycling. XRD patterns of c) B@TC and d) P@TC before and after repeated heating/cooling thermal cycling (SL46 as a control sample).

**
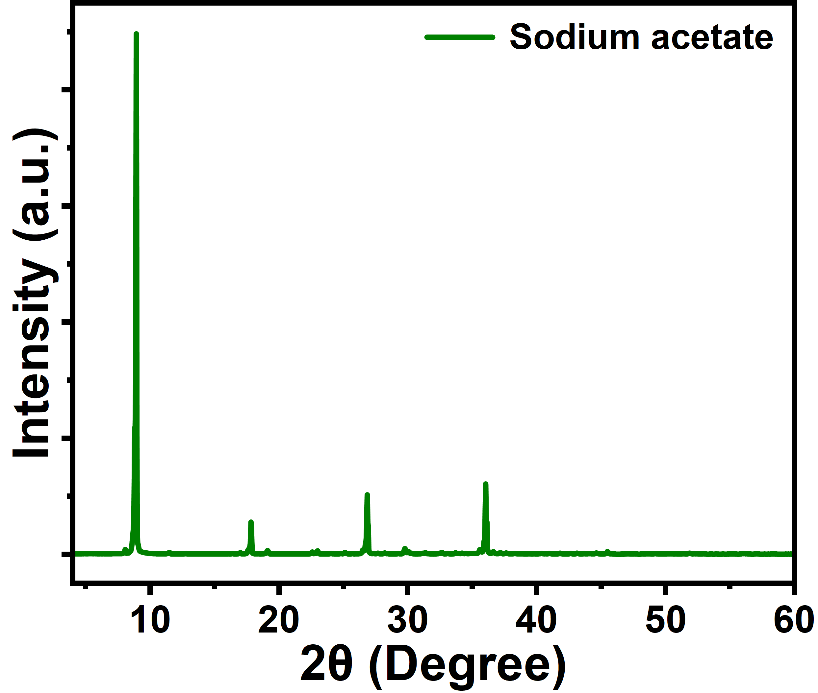
**

**Figure S16.** XRD pattern of SAT without crystalline water.

**
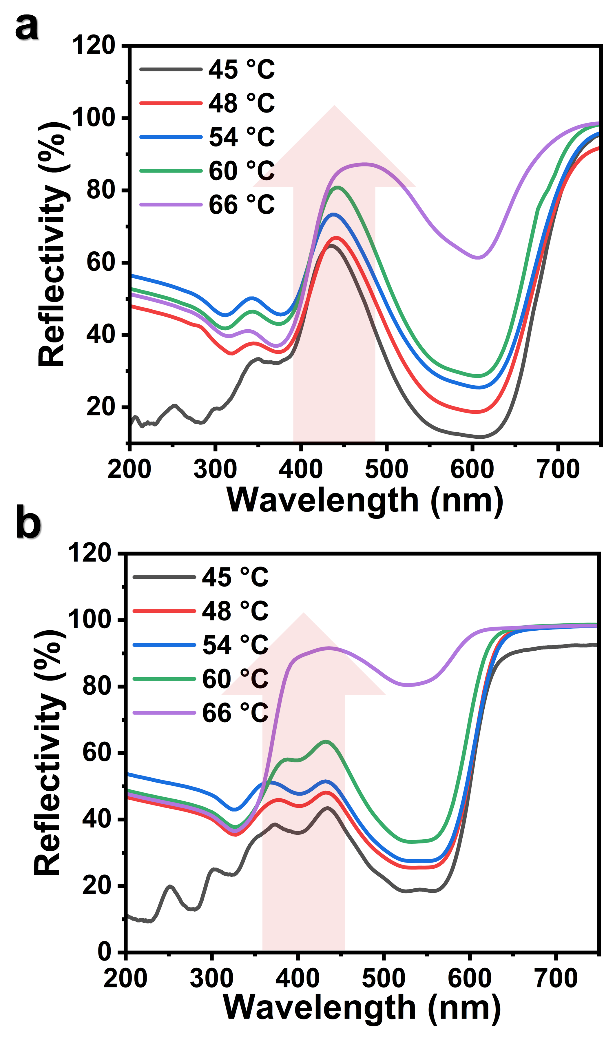
**

**Figure S17.** UV-visible spectra of B@TC and P@TC at different temperatures.

**
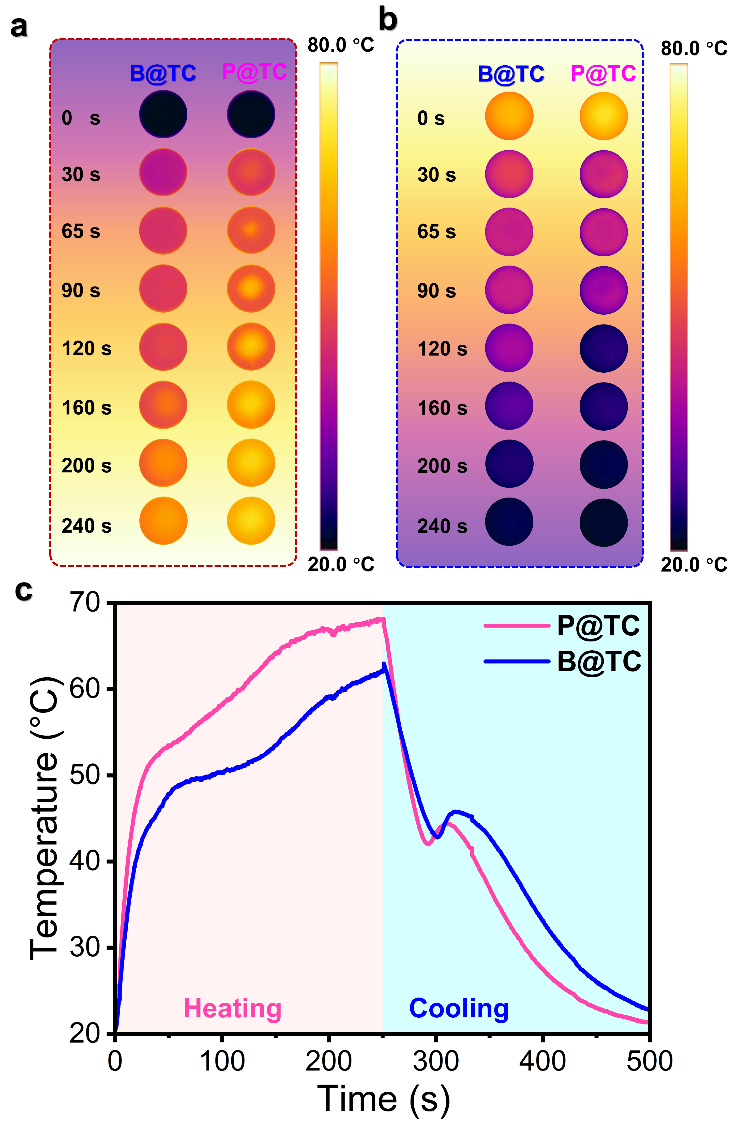
**

**Figure S18.** Representative infrared thermal images of B@TC and P@TC in the a) heating and b) cooling processes. c) Temperature evolutions of B@TC and P@TC in the heating and cooling processes.

**
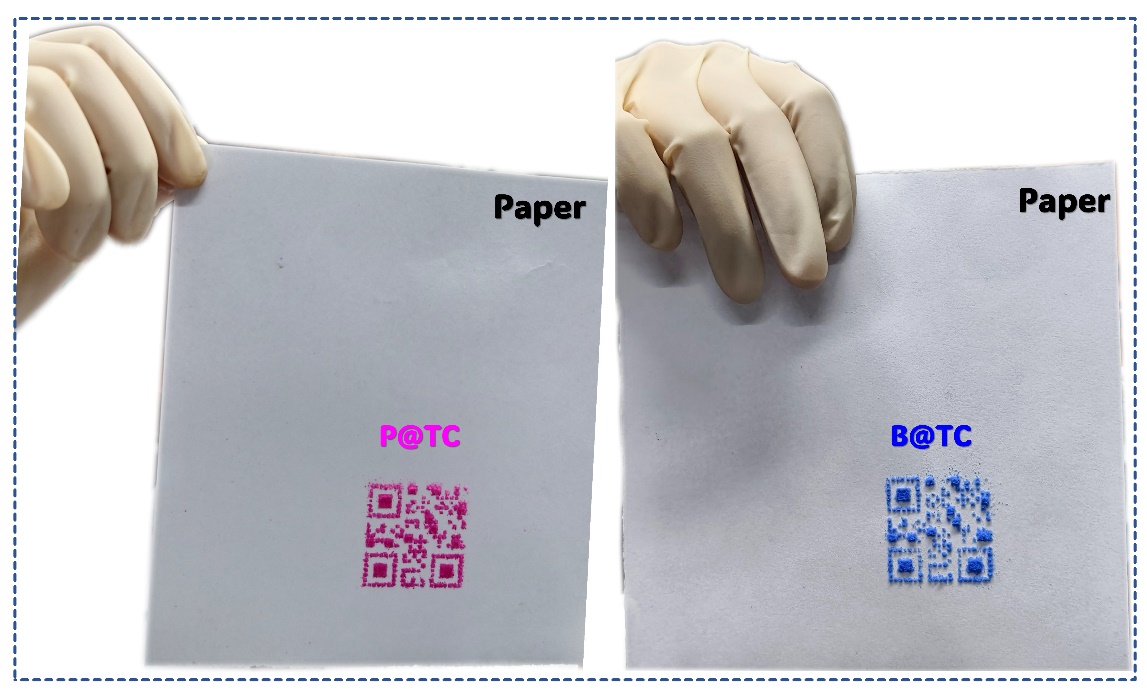
**

**Figure S19.** Digital photographs of thermochromic QR code labels fabricated using (a) B@TC and (b) P@TC composites, adhered to paper substrates. These labels demonstrate the physical form prior to temperature-dependent activation.

**Table S1.** Parameters of SAT/LA simulation model constructed with a molar ratio of SAT:LA=48:49.

| Lattice length (*a*) | Lattice length (*b*) | Lattice length (*c*) | Lattice angle (*α*) | Lattice angle (*β*) | Lattice angle (*γ*) |
| --- | --- | --- | --- | --- | --- |
| 46.2110 Å | 34.0740 Å | 39.1638 Å | 91.0414° | 155.1050° | 90.8444° |

**Table S2.** Thermal parameters of SAT and LA used in the Schroder-Van Laar equation.

| Sample | *T*_m_/K | Δ*H*_m_/J g^‒1^ | M/g mol^‒1^ |
| --- | --- | --- | --- |
| SAT | 334.95 | 37408.4 | 136.08 |
| LA | 317.15 | 34615.0 | 200.32 |

**Table S3.** Comparison of the developed SL46 with other representative shape-stabilized PCM composites used in the thermochromic systems.

| Type  of PCM | Stabilization method | Stabilization process | Latent heat  (J g^−1^) | Leakage rate | Cycling stability | Cost of PCM | Cost of stabilizer | Reference |
| --- | --- | --- | --- | --- | --- | --- | --- | --- |
| Ethyl stearate | Porous filter paper composite | Vacuum-assisted impregnation | 75.36 | No leakage | Δ*H*_m_ decreases  to 67.55 J g^−1^  (100 cycles) | 153.44 $ kg^−1^ | 4.85 $ kg^−1^ | [8] |
| Butyl stearate | Porous filter paper composite | Vacuum-assisted impregnation | 55.63 | No leakage | Stable  (100 cycles) | 177.24 $ kg^−1^ | 4.85 $ kg^−1^ | [8] |
| Butyl stearate | Microencapsulation | In-situ  polymerization | 122.20 | No leakage | Stable  (100 cycles) | 177.24 $ kg^−1^ | 144.15 $ kg^−1^ | [9] |
| Tetradecyl alcohol | 3D gel composite | Physical  adsorption | 196.10 | No leakage | Not reported | 22.48 $ L^−1^ | 190.78 $ kg^−1^ | [10] |
| PEG | Chemical Cross-linking | Dynamic covalent cross-linking | 98.50 | No leakage | Not reported | 13.77 $ kg^−1^ | 144.72 $ kg^−1^ | [11] |
| Methyl Laurate | Microencapsulation | Complex  coacervation | 110.35 | No leakage | Stable  (10 cycles) | 56.20 $ L^−1^ | 128.70 $ kg^−1^ | [12] |
| Dodecanol | Microencapsulation | Interfacial Polymerization | Not reported | < 3.1%  (below 100°C) | Not reported | 29.51 $ L^−1^ | 138.39 $ kg^−1^ | [13] |
| 1-Octadecanol | Microencapsulation | In-situ  polymerization | Not reported | Not reported | Stable  (16 cycles) | 27.54 $ kg^−1^ | 86.55 $ kg^−1^ | [14] |
| 1-Hexadecanol | Microencapsulation | Complex coacervation and dual crosslinking | 56.5−80.7 | Not reported | Packaging efficiency decreases by 4.5%  (100 cycles) | 10.64 $ kg^−1^ | 168.04 $ kg^−1^ | [15] |
| 1-Tetradecanol | Microencapsulation | In-situ  polymerization | 200.00 | Not reported | Stable  (100 cycles) | 22.48 $ kg^−1^ | 73.34 $ kg^−1^ | [16] |
| Methyl behenate | Microencapsulation | Emulsion and in-situ polymerization | 200.20 | No leakage | 99.0% enthalpy retention  (100 cycles) | 76.71 $ kg^−1^ | 73.34 $ kg^−1^ | [17] |
| Lauric acid | Blending | Solution blending | 151.9 | No leakage | Δ*H*_m_ decreases  to 145.9  (100 cycles) | 24.73 $ kg^−1^ | 323.45 $ kg^−^ | [18] |
| SL46 | Eutectic | Blending | 169.8 | No leakage | Stable (100 cycles) | 7.95 kg^−1^ | 7.95 kg^−1^ | This work |

Note: The costs of PCMs and stabilizers are based on the price listed in Shanghai Macklin Biochemical Co., Ltd., China.

**REFERENCES**

[1] a) H. Zhang, S. Zhou, H. Liu, Z. Qian, X. Wang, *Adv. Funct. Mater.* **2024**, *34*, 2408269; b) M. Sun, T. Liu, M. Li, T. Liu, X. Wang, G. Chen, D. Jiang, *J. Energy Storage* **2023**, *62*, 106956.

[2] Z. Liu, X. Jin, Y. Li, J. Jiang, *J. Energy Storage* **2025**, *111*, 115466.

[3] K. Wood, A. Froelich, A. Paciaroni, M. Moulin, M. Haertlein, G. Zaccai, D. J. Tobias, M. Weik, *J. Am. Chem. Soc.* **2008**, *130*, 4586.

[4] B. Wang, H. Gao, H. Wu, Y. Wu, B. Ren, X. Liu, Y. Nie, *Chem. Eng. J.* **2024**, *488*, 151175.

[5] Y. Mao, J. Li, X. Yang, K. Tao, K. Sun, S. Chen, Y. Zheng, *J. Energy Storage* **2024**, *94*, 112104.

[6] G. Xia, Z. Luo, Y. Yang, L. Chen, R. Li, C. Du, Y. Yu, *Int. J. Heat Mass Transfer* **2024**, *192*, 107249.

[7] M. Sun, L. Lin, H. Di, Y. Feng, *Int. J. Heat Mass Transfer* **2025**, *244*, 126932.

[8] H. Xu, L. Jiang, A. Yuan, Z. Wei, Y. Lei, Y. Wang, W. Kong, X. Fu, J. Lei, *J. Energy Storage* **2022**, *50*, 104292.

[9] S. Liu, H. Zhang, X. Zhang, W. Li, *J. Energy Storage* **2025**, *119*, 116329.

[10] S. Zhao, A. Yuan, X. Chen, Y. Lei, X. Fu, J. Lei, L. Jiang, *Chem. Eng. J.* **2024**, *481*, 148698.

[11] A. Yuan, S. Zhao, T. Liu, Y. Zhao, L. Jiang, J. lei, *Adv. Mater. Technol.* **2022**, *7*, 2200226.

[12] Y. Pu, J. Fang, *Colloids Surf., A* **2022**, *653*, 129889.

[13] X. Guo, S. Daka, M. Fan, X. Lin, W. Sun, *J. Mater. Sci.* **2023**, *58*, 2188.

[14] X. Wang, X. Wang, Y. Cui, *Mater. Chem. Phys.* **2023**, *305*, 127901.

[15] B. Wu, L. Shi, Q. Zhang, W.-J. Wang, *RSC Adv.* **2017**, *7*, 42129.

[16] Q. Liu, S. Gao, G. Wang, *J. Energy Storage* **2025**, *134*, 118274.

[17] J. Wu, P. Yu, L. Wang, X. Zhang, W. Xie, J. Zeng, G. Zhou, Z. Zhang, *small* **2025**, *21*, 2505575.

[18] Z. Li, Y. Zhang, X. Wang, F. Cao, X. Guo, S. Zhang, B. Tang, *J. Power Sources* **2024**, *603*, 234447.
